# Daily Sleep Quality and Support in Romantic Relationships: The Role of Negative Affect and Perspective-Taking

**DOI:** 10.1007/s42761-023-00180-7

**Published:** 2023-03-03

**Authors:** Nicole T. Sell, Natalie M. Sisson, Amie M. Gordon, Sarah C. E. Stanton, Emily A. Impett

**Affiliations:** 1 University of Toronto Mississauga, Mississauga, Canada; 2 University of Michigan, Ann Arbor, USA; 3 University of Edinburgh, Scotland, UK

**Keywords:** Sleep, Support, Negative Affect, Perspective-taking, Close Relationships

## Abstract

Sleep is an important predictor of social functioning. However, questions remain about how impaired sleep—which is common and detrimental to affective and cognitive functions necessary for providing high quality support—is linked to both the provision and perception of support, especially at the daily level. We tested links between impaired sleep and provided and perceived support in romantic couples, and whether these links were mediated by negative affect and perspective-taking. In preregistered analyses of two 14-day diary studies (Study 1 *N* = 111 couples; Study 2 *N* = 100 couples), poor daily subjective sleep quality—but not duration—was associated with less self-reported support toward a partner (in both studies), less perceived support from a partner and less partner-reported support (in Study 1), and partner perceptions of receiving less support (in Study 2). Only greater daily negative affect consistently mediated the association between participants’ impaired sleep (i.e., poor subjective sleep quality and duration) and their own support provision, as well as their partner’s perceptions of received support. Our findings suggest that the effect of sleep on social processes may be strongest for self-reported measures of support and that unique aspects of sleep might be differentially associated with social outcomes given that sleep quality—but not duration—was consistently linked to support outcomes. These findings highlight the psychosocial influences of sleep and negative affect, and may inform approaches to promote supportive partner interactions.

**Supplementary Information:**

The online version contains supplementary material available at 10.1007/s42761-023-00180-7.

Sleep problems are common and pervasive, with 69% of adults in the USA getting less sleep than they need (National Sleep Foundation, 2014) and 33–52% frequently waking up feeling unrested (National Center for Health Statistics, 2016). Impaired sleep—defined here as fatigue, lower subjective sleep quality, or shorter sleep duration—can detract from cognitive, behavioral, and affective skills that are crucial for providing and perceiving support during social interactions. In turn, decrements to these skills can limit positive interpersonal interactions and general well-being (Engle-Friedman & Young, 2019; Gordon et al., 2017, 2021). Despite growing attention paid to the association between sleep and social support (e.g., Kent de Grey et al., 2018; i.e., an individual’s responsiveness to the needs of close others; Taylor, 2011), surprisingly few studies have simultaneously examined the effects of sleep on the support people *provide* to and *perceive* from close others. This is problematic as the amount of support given by a provider is only weakly related to the amount of support perceived by the recipient (Haber et al., 2007). As such, impaired sleep may undermine support provision and perception in nuanced ways not captured by examining only one aspect of support.

Given that romantic partners may disturb each other’s sleep by sharing a bed (e.g., through snoring) and are often one another’s primary source of support (Umberson et al., 2010), the association of sleep with support provision and perception is especially pertinent within intimate relationships. Romantic partners provide daily opportunities for positive and negative interpersonal interactions that can be influenced by the cognitive and affective impairments associated with impaired sleep (Gordon & Chen, 2014; Hasler & Troxel, 2010; Maranges & McNulty, 2017). Although research highlights links between sleep and social support, few studies have tested daily (within-person) effects of poor sleep (i.e., when people sleep worse or less than they usually do) on both provided and perceived partner support, nor have past studies thoroughly investigated the cognitive and affective pathways potentially underlying these links. Considering that support is a cornerstone of romantic relationships (Collins et al., 2010), addressing these identified open questions is crucial. Thus, the current research assesses daily effects of sleep on both provided and perceived partner support and potential affective and cognitive mediators of these associations.

Extant research provides evidence of a sleep-support link among middle-aged and older couples in well-established relationships. Specifically, impaired sleep has been associated with less self-reported emotional support provided to one’s romantic partner (Kane & Krizan, 2021) and the receipt of less frequent spousal support over a 10-day period (Yorgason et al., 2018). Although research with younger couples—who may have different sleep (Grandner, 2012; Moraes et al., 2014) and support (Umberson et al., 2010) experiences—is needed before drawing definitive conclusions, it nonetheless appears that impaired sleep may hinder provided and perceived partner support.

Regarding mediators, impaired sleep has been consistently linked to greater negative affect, such as increased anger (McCrae et al., 2008; Moturu et al., 2011; Ong et al., 2011). Greater negative affect has, in turn, been linked to less self-reported emotional and instrumental partner support provision (e.g., Devoldre et al., 2010; Iida et al., 2010). Furthermore, a study of older couples found that wives’ lower daily enthusiasm following impaired sleep was associated with wives’ lower perceived frequency of support *from* their partner through wives’ greater reported negative mood (Yorgason et al., 2018), suggesting that impaired sleep may also make it more difficult for people to be supported by their partners. Importantly, this study exclusively examined perceived support *frequency*, making it unclear if the pattern would be similar for provided support or for support *quality*, which is critical for relationship outcomes and support effectiveness (Rini & Dunkel-Schetter, 2010; Zee et al., 2020). Therefore, given that older adults often experience less dramatic increases in negative affect following poor sleep relative to younger adults (Schwarz et al., 2019), it is necessary to assess support quality and test affective pathways for both provided and perceived support among younger couples.

Although no work has tested perspective-taking (i.e., the ability to understand another’s perspective) as a mediator, it has been independently linked to both sleep and support (e.g., Deliens et al., 2018; Devoldre et al., 2010). Experimentally, impaired sleep is associated with diminished cognitive and visual perspective-taking task performance (Deliens et al., 2015, 2018) and lower empathic accuracy between romantic partners following conflict conversations (Gordon & Chen, 2014). Together, these findings suggest that poor sleep may undermine the ability to adopt other people’s perspectives. Given that high-quality support requires the capacity to be attentive and responsive to close others’ needs (Feeney & Collins, 2015), experiencing sleep-related impairments in the ability to understand the perspective of one’s partner would likely undermine the capacity to provide and perceive partner support. Indeed, both theory and empirical investigations have linked perspective-taking to support provision in close relationships (Devoldre et al., 2010; Feeney & Collins, 2015; Verhofstadt et al., 2016). For example, lower perspective-taking has been linked to the provision of less emotional and instrumental partner support among young and middle-aged adults (Devoldre et al., 2010; Verhofstadt et al., 2016). While no studies to our knowledge have tested the proposed mediation model, impaired sleep may undermine cognitive resources important for perspective-taking that, in turn, may impair the ability to provide and perceive effective partner support.

## The Current Research

The main goal of this research was to provide empirical tests of the within-person links between impaired sleep and social support (provided and perceived) among young couples using ecologically valid daily diary paradigms by separating these links from between-person associations. A secondary goal was to examine affective (negative emotions) and cognitive (perspective-taking) mediators that may explain the proposed sleep-support links. Consistent with extant research on sleep and support among older couples, we had the following set of preregistered hypotheses (accessible at https://osf.io/j8kce/). First, we predicted that when people reported experiencing more impaired sleep (i.e., shorter or poorer quality sleep) than usual, they would report (1) providing less support to and (2) receiving less support from their partner. Second, given that impaired sleep may cause people to exhibit negative affect that makes effective support difficult for others to provide and perceive, we expected that when people reported experiencing more impaired sleep, their *partners* would report (3) providing less support to and (4) receiving less support from them (i.e., the impaired sleepers). We additionally predicted that when people reported experiencing more impaired sleep than usual, they would (5) report providing less support to their partner and (6) have partners who perceived them as providing less support via the impaired sleepers’ greater reported negative affect and lower reported and perceived perspective-taking.

## Study 1: Method

### Participants

Our sample consisted of 111 couples (*N* = 222), the majority of which we presume to be heterosexual, although sexual orientation was not assessed. Participants were recruited from the Greater Toronto Area through online advertisements (e.g., on Kijiji.com) and community outreach for a broader project investigating romantic partners’ interactions. We selected our sample size based on available resources and other studies that examined associations between predictors and relationship outcomes in romantic couples with naturalistic diary methods around the time of data collection (2015–2016; 46 couples in Kane et al., 2014; 108 couples in Kelly & Bagley, 2017; 68 couples in Maranges & McNulty, 2017). Our target sample size was 100 couples, and we retained an additional 11 couples that were already scheduled to participate in order to compensate for any potential missing data at the end of the study. However, sensitivity analyses using the simr R package (Green & McLeod, 2016; based on the average number of days participants responded to sleep and support items, which was 11) indicated that we had 80% power to detect small level 1 effect sizes (*R*^2^ = 0.06) and medium level 2 effect sizes (*R*^2^ = 0.19). According to these conservative power simulations and recent guidelines (Arend & Schäfer, 2019), we are underpowered to detect small between-person effects. As such, we focus on within-person effects throughout.

Participants were eligible to participate if they were 18 years or older and had been in a romantic relationship for at least one year, though many were in longer relationships (*M* = 4.13 years, *SD* = 2.67). Our sample ranged in age from 18 to 57 years old (*M* = 26.76, *SD* = 7.17; 48.2% women, 49.5% men, and 2.5% other [e.g., preferred not to say]). Most participants (74.77%) were in committed unmarried relationships, 22.97% were married, and 2.25% did not report their relationship status. The sample was also ethnically diverse and included participants with the following self-reported backgrounds: 24.63% Western European, 18.23% South Asian, 7.88% Eastern European, 6.90% Caribbean, 5.42% South American, 2.46% African, 2.46% Middle Eastern, 2.46% Southeast Asian, 12.29% bi- or multi-ethnic, 12.81% other, and 2.46% unreported.

### Procedure

Participants completed a multi-part study^1^ investigating how romantic couples resolve conflicts of interest that consisted of a baseline questionnaire, an in-lab session, and a 14-day daily diary. Both partners began the study by completing a 1-h online questionnaire that assessed sample demographics, including relationship status, relationship length, and cohabitation status. After completing the baseline questionnaire, each participant received $15 CAD as monetary compensation and began the 14-day daily diary. As per our preregistration, we also tested our hypotheses with similar items during the in-lab session. However, we found low means and variability among our self-reported and rater-observed in-lab negative affect items, suggesting that there may have been little need for support in this contrived context. As such, the laboratory procedure and results are reported in the Supplementary Online Materials.

Both partners were independently emailed a questionnaire at 6:00 pm each evening and given until 12:00 am to answer questions that assessed their experiences and behaviors that day, including their negative affect. Moreover, on days when couples did not report a conflict of interest (which was a primary interest in the larger project), participants answered additional questions that measured their subjective sleep quality and duration, perspective-taking, perceptions of their partner’s perspective-taking, provision of partner support, and perceptions of their partner’s provision of support. These days without conflicts of interest comprised 71% of all diary days, or an average of 11 out of 14 days per person. In addition, while participants completed all survey questions at the same time, they were nonetheless asked to reflect on different parts of their day when answering questions about our variables of interest (e.g., they were asked about their negative affect throughout the day, but were specifically asked to reflect on their sleep quality upon waking up). At the end of the 2 weeks, participants were compensated up to $40 CAD (prorated based on the number of daily surveys completed).

### Daily Measures

#### Sleep Quality

Participants rated their daily subjective sleep quality while reflecting on how they felt when they woke up (“When I woke up today, I felt: _____?”) on a 4-point scale (1 = *refreshed*, 4 = *fatigued*; *M* = 2.37, *SD* = 0.98) and sleep duration (“I slept a total of ____ hours?”) using an open-ended response option (*M* = 7.21, *SD* = 1.72). Importantly, given that theory (Buysse, 2014) and empirical findings (Gordon et al., 2021) suggest that different dimensions of sleep may have unique effects on social processes and health outcomes, we deviated from our preregistration by examining the separate effects of subjective sleep quality and sleep duration on support provision (see the Supplementary Online Materials for analyses utilizing a more holistic sleep composite).

#### Perspective-Taking

Participants rated their own daily perspective-taking (“Today, I was able to take my partner’s perspective and understand what s/he was thinking and feeling”) on a 7-point scale (1 = *not at all*, 7 = *a lot; M* = 4.94, *SD* = 1.56) and their perceptions of their partner’s daily perspective-taking (“Today, my partner was able to take my perspective and understand what I was thinking and feeling”) on a 7-point scale (1 = *not at all*, 7 = *a lot*; *M* = 4.77, *SD* = 1.65).

#### Negative Affect

Participants rated the degree to which they felt three negative emotions over the course of the day (“How much did you feel anxious/stressed/nervous,’ ‘sad,’ and ‘angry’ today”) on a 7-point scale (1 = *not at all*, 7 = *a lot*), which we averaged into a negative affect composite (*M* = 2.32, *SD* = 1.32). Within-person reliability of these items (indicated by *R*_*C*_; Bolger & Laurenceau, 2013) was 0.68.

#### Partner Support Provision

Participants rated the overall daily support they provided to their partner (“I met my partner’s needs today”) on a 7-point scale (1 = *not at all*, 7 = *a lot*; *M* = 4.42, *SD* = 1.72). They also rated the overall perceived daily support from their partner (“My partner met my needs today”) on a 7-point scale (1 = *not at all*, 7 = *a lot*; *M* = 4.67, *SD* = 1.65).

## Study 1: Results

### Analysis Overview

Following recommendations for dyadic longitudinal data analysis, we conducted two-level models in which partners’ residuals were nested within couples and time and random intercepts were modeled for each partner within couples (rather than time within individuals; Bolger & Laurenceau, 2013; Kashy & Donnellan, 2018). We modeled the non-independence between partners’ level 1 residuals within couples and days in order to adjust for the non-independence between partners. Dyads were treated as indistinguishable such that fixed and random estimates were pooled across partners within couples. Because the partners were treated as indistinguishable, we used a compound symmetry structure for the variance–covariance matrix, constraining the two partners to have the same variance. We note here that we did not model the autoregressive structure across days given the constraints of our software (i.e., these cannot be modeled for crossed residuals in dyadic data).

Our analytic models were also guided by the actor-partner interdependence model (APIM; Cook & Kenny, 2005). Specifically, given that romantic partners’ sleep quality and duration are likely correlated because they share a bed and may influence each other’s sleep (e.g., through snoring, setting an alarm clock), we simultaneously included both partners’ sleep variables as predictors to test our main effects and moderations in IBM SPSS Version 26. This allowed us to see how one partner’s impaired sleep is uniquely related to their own reports of support and their partner’s reports of support, which is important given that the support people actually provide may not align with the support they think they provide. In order to isolate the unique within-person effects of sleep on social support, we first created both within-person and between-person predictors by aggregating our daily diary predictor variables (i.e., averaging across days within each person), which were subtracted from the original scores to create person-mean centered predictors and subsequently grand-mean centered to create between-person predictors. We then entered both our within-person and between-person predictors for each partner into the same model to generate estimates for unique within-person and between-person links to our outcome variables.

In accordance with our preregistration, we used the Monte Carlo Method for Assessing Mediation (MCMAM) to estimate the indirect effects of sleep on support through negative affect and perspective-taking. Given that models without random slopes do not need to account for covariance between the *a* and *b* paths in mediation models changing as a result of random slopes, we deviated from our preregistration and did not also use MLMED to test these models. As such, we do not report any mediation results run using MLMED in the main text and only report our MCMAM results (see the Supplementary Online Materials for more information). Given that it is not recommended to enter multiple mediators into the same model when they may be correlated (Kenny et al., 2003), we preregistered that we would test mediators separately and only include both mediators in the same model when both mediators were significant. However, correlations between negative affect and perspective-taking in both studies were low (see Table 1). Thus, we deviated from our preregistration to enter both mediators into the model simultaneously, allowing us to reduce the number of statistical tests and evaluate the unique predictive ability of each mediator (see the Supplementary Online Materials for original models with only one mediator). We evaluated these models using bootstrapped 95% confidence intervals based on 20,000 samples (Selig & Preacher, 2008). Confidence intervals that did not include 0 were considered significant.

**Table 1:** Within-person correlations among diary variables (Study 1)

	1	2	3	4	5	6	7	8	9	10	11
1. Subjective sleep quality	–										
2. Sleep duration	.45**	–									
3. Provided support (self-reported)	.08**	− .02	–								
4. Received support (self-reported)	.08**	.00	.68**	–							
5. Provided support (partner-reported)	.07*	.01	.27**	.25**	–						
6. Received support (partner-reported)	.05	− .03	.25**	.22**	.68**	–					
7. Negative affect (self-reported)	− .17**	− .08**	− .22**	− .20**	− .13**	− .12**	–				
8. PT (self-reported)	.09**	.02	.38**	.42**	.15**	.14**	− .15**	–			
9. Perceived PT (self-reported)	.10**	.04	.38**	.48**	.15**	.17**	− .18**	.68**	–		
10. PT (partner-reported)	.03	.00	.15**	.14**	.38**	.42**	− .06*	.09**	.15**	–	
11. Perceived PT (partner-reported)	.03	− .00	.15**	.17**	.38**	.48**	− .06*	.15**	.21**	.68**	–

*PT,* perspective-taking. Within-person correlations were calculated in R version 4.1.1 (R Core Team, 2020) with the rmcorr (Bakdash & Marusich, 2021) package. *p < .05, **p < .01

Importantly, given that our independent variables, mediators, and dependent variables all had variability at the within-person and between-person level, we were subsequently able to estimate within-person and between-person indirect effects (in order to isolate unique within-person effects) using MCMAM. Within-person correlations among the variables—which account for nesting—are presented in Table 1.

### Main Effects of Impaired Sleep on Support Provision

We predicted that people who reported more impaired sleep would report both *providing* less support to their partner and *perceiving* less support from their partner in their everyday lives. Similarly, we predicted that the partners of people who reported more impaired sleep would also report providing less support to the person who slept poorly and perceive less support from them. Although we analyzed both within-person and between-person effects of sleep, given the large number of analyses and our greater power to detect within-person effects, we focus on within-person effects when reporting the results below and present between-person effects in the Supplementary Online Materials. Given that we analyzed subjective sleep quality and sleep duration separately, we tested our hypotheses accordingly and will present these results in turn, starting with subjective sleep quality.

#### Subjective Sleep Quality

In line with our predictions, participants’ daily subjective sleep quality (i.e., their subjective sleep quality on a given night compared to their own average subjective sleep quality) was associated with their own support provision and their perceptions of the support they received from their partner (see Table 2 for all subjective sleep quality results).

**Table 2:** Within-person effects of subjective sleep quality and sleep duration on support provision (Study 1)

Predictors	*b*	*SE*	*df*	*t*	*p*	*R*^2^
Provided support (self-reported)						
Sleep quality	0.11*	0.05	1037.40	2.34	.02	0.002
Sleep duration	− 0.01	0.03	1067.47	− 0.23	.82	–
Provided support (partner-reported)						
Sleep quality	0.11*	0.05	1037.56	2.23	.03	0.002
Sleep duration	0.01	0.03	1068.23	0.34	.74	–
Received support (self-reported)						
Sleep quality	0.11*	0.05	1037.97	2.36	.02	0.002
Sleep duration	0.02	0.02	1057.10	0.36	.36	–
Received support (partner-reported)						
Sleep quality	0.07	0.05	1038.55	1.55	.12	–
Sleep duration	− 0.02	0.03	1055.43	− 0.98	.32	–

*p < .05

Specifically, when participants felt more fatigued than usual upon waking, they reported providing less support to their partner and perceiving less support from their partner that day. Partners also reported providing less support, corroborating the association between fatigue and participants’ perceptions of receiving less support, but did not report receiving less support themselves when their partners (i.e., the poor sleepers) were more fatigued than usual.

#### Sleep Duration

Participants’ daily sleep duration (i.e., their sleep duration on a given night compared to their own average sleep duration) was not significantly associated with any of the outcome variables (see Table 2).

### Mediating Effects of Negative Affect and Perspective-Taking on Impaired Sleep and Support

**Fig. 1:**
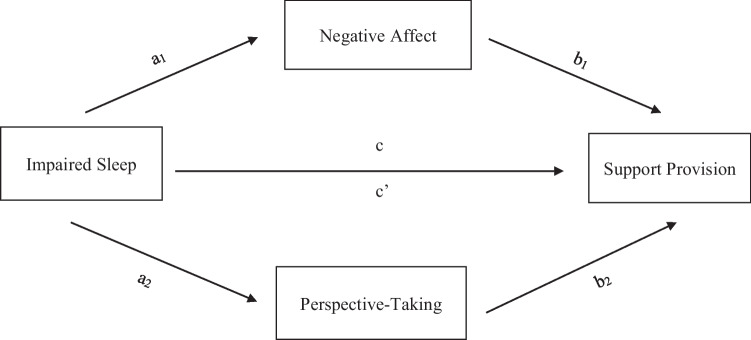
Sample mediation model of the association between impaired sleep and support provision through negative affect and perspective-taking

We next examined the hypothesis that the associations between impaired sleep and self-reported and partner-perceived support provision would be mediated by poor sleepers’ negative affect and perspective-taking (self-reported and partner-perceived). In line with previous work (e.g., MacKinnon et al., 2007), we tested our preregistered mediation models even in cases in which the main effect was null to assess if there was evidence of an indirect effect (see Table 3 for all within-person model statistics controlling for partner’s sleep and the Supplementary Online Materials for between-person effects). We also conducted additional exploratory analyses (which we preregistered in Study 2) to investigate mediations with perceived partner perspective-taking included in our models. Both mediators (i.e., negative affect and self-reported perspective-taking, negative affect and perceived partner perspective-taking, negative affect and partner-perceived perspective-taking) were entered into the same model in order to assess their unique effects (see Fig. 1 for a sample mediation model that visualizes our general predictions).

**Table 3:** Within-person effects of subjective sleep quality and duration on support mediated by negative affect and perspective-taking (Study 1)

Predictors	Mediators	*a*	*b*	*ab*	*c*	*c’*	95% CI	
							Lower	Upper
Provided support (self-reported)								
Sleep quality	NA (and self-reported PT)	− 0.20***	− 0.19***	0.06	0.11*	0.05	0.02	0.06
Sleep duration	NA (and Self-Reported PT)	− 0.06**	− 0.19***	0.01	− 0.01	− 0.02	0.004	0.02
Sleep quality	NA (and own perceived PT)	− 0.21***	− 0.18***	0.04	0.11*	0.03	0.02	0.06
Sleep duration	NA (and own perceived PT)	− 0.07***	− 0.18***	0.01	− 0.01	− 0.02	0.005	0.02
Sleep quality	NA (and partner-perceived PT)	− 0.22***	− 0.24***	0.05	0.11*	0.05	0.03	0.08
Sleep duration	NA (and partner-perceived PT	− 0.07***	− 0.25***	0.03	− 0.01	− 0.02	0.01	0.03
Sleep quality	Self-reported PT (and NA)	0.07^†^	0.38***	0.03	0.11*	0.05	− 0.05	0.06
Sleep duration	Self-reported PT (and NA)	0.01	0.38***	0.004	− 0.01	− 0.02	− 0.01	0.02
Sleep quality	Own perceived PT (and NA)	0.09^†^	0.37***	0.03	0.11*	0.03	− 0.001	0.07
Sleep duration	Own perceived PT (and NA)	0.02	0.37***	0.01	− 0.01	− 0.01	− 0.01	0.03
Sleep quality	Partner-perceived PT (and NA)	0.04	0.10**	0.004	0.11*	0.05	− 0.005	0.02
Sleep duration	Partner-perceived PT (and NA)	− 0.04	0.09*	− 0.004	− 0.01	− 0.02	− 0.01	0.004
Received support (partner-reported)								
Sleep quality	NA (and self-reported PT)	− 0.20***	− 0.08*	0.02	0.07	0.04	0.0004	0.03
Sleep duration	NA (and self-reported PT)	− 0.06**	− 0.09*	0.01	− 0.02	− 0.03	0.001	0.01
Sleep quality	NA (and own perceived PT)	− 0.21***	− 0.08^†^	0.02	0.07	0.03	− 0.0004	0.04
Sleep duration	NA (and own perceived PT)	− 0.07***	− 0.09*	0.01	− 0.02	− 0.03	0.001	0.01
Sleep quality	NA (and partner-perceived PT)	− 0.22***	− 0.09*	0.02	0.07	0.03	0.004	0.04
Sleep duration	NA (and partner-perceived PT)	− 0.07***	− 0.10**	0.01	− 0.02	− 0.03	0.002	0.01
Sleep quality	Self-reported PT (and NA)	0.07^†^	0.08*	0.01	0.07	0.04	− 0.001	0.02
Sleep duration	Self-reported PT (and NA)	0.01	0.08*	0.001	− 0.02	− 0.03	− 0.002	0.01
Sleep quality	Own perceived PT (and NA)	0.09^†^	0.13***	0.01	0.07	− 0.0005	– 0.0002	0.03
Sleep duration	Own perceived PT (and NA)	0.02	0.13***	0.003	− 0.02	− 0.04	− 0.003	0.01
Sleep quality	Partner-perceived PT (and NA)	0.04	0.49***	0.02	0.07	0.03	− 0.02	0.07
Sleep Duration	Partner-perceived PT (and NA)	− 0.04	0.49***	− 0.02	− 0.02	− 0.03	− 0.03	0.02

*NA*, negative affect. *PT*, perspective-taking. ^†^*p* < .10, *p < .05, **p < .01, ***p < .001

#### Subjective Sleep Quality

Consistent with our predictions, participants’ daily negative affect accounted for the direct association between their daily subjective sleep quality and their own support provision when also accounting (separately) for self-reported and perceived perspective-taking. Participants’ daily negative affect additionally accounted for the indirect association between their daily subjective sleep quality and their partner’s perceptions of the support provision they received when also accounting (separately) for self-reported and partner-perceived perspective-taking, but not when accounting for participant’s perceptions of their partner’s perspective-taking. These findings generally indicate that when people felt more fatigued than usual, they reported experiencing greater negative affect that day and, in turn, reported providing less support to their partner and had partners who reported receiving less support from them. In these same models, however, all confidence intervals for within-person reported and perceived perspective-taking as a mediator contained zero and, as such, were not significant (see Table 3).

#### Sleep Duration

Similarly, participants’ daily negative affect accounted for the indirect association between their daily sleep duration and their own support provision, as well as the indirect association between participants’ daily sleep duration and their partner’s perceptions of the support provision they received when also accounting (separately) for self-reported and perceived perspective-taking. These findings indicate that when people slept less than normal the previous night, they reported experiencing greater negative affect that day and, in turn, reported providing less support to their partner and had partners who reported receiving less support from them. In these same models, however, all confidence intervals for within-person reported and perceived perspective-taking as a mediator contained zero and, as such, were not significant (see Table 3).

Demonstrating further evidence for our proposed pathways, we conducted reverse mediation models in which we tested if impaired sleep predicted negative affect and perspective-taking through own self-reported and partner-perceived support. In line with the notion that negative affect and perspective-taking precede support provision, all confidence intervals for within-person self-reported and partner-perceived support as a mediator contained zero and, as such, were not significant (see Tables 21 and 22 in the Supplementary Online Materials for all reverse mediation confidence intervals).

### Generalizability of Findings

**Fig. 2:**
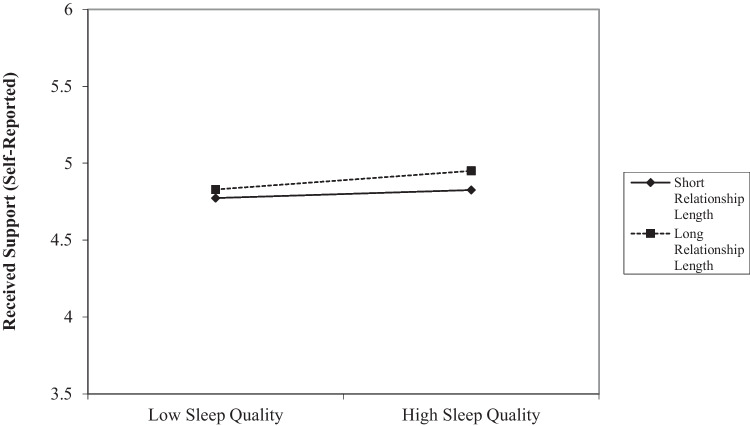
The effects of within-person sleep duration on received support (self-reported) moderated by relationship length (Study 1). *Note.* Low values represent 1 SD below the mean, high values represent 1 SD above the mean

In a final set of analyses, we tested whether all the main effect models described above (which included both partners’ sleep) were moderated by participants’ gender or relationship length to examine the robustness of our findings. Specifically, we conducted moderations separately for subjective sleep quality and duration, but included within- and between-person predictors for both partners within each model (e.g., both partners’ within-person and between-person subjective sleep quality were entered as simultaneous predictors). We assessed each outcome in a separate model, with one moderator at a time (i.e., gender and relationship length were assessed in separate moderation models). Our results revealed that relationship length moderated the within-person association between participants’ sleep duration and their perceptions of the support provision they received (see Fig. 2). Among participants who had been in their relationship for a shorter duration of time, sleeping less than usual on a given day was not significantly associated with their perceptions of received support. In contrast, participants who had been in their relationship for a longer duration of time reported receiving less support from their partner when they slept less than they usually did on a given day. This was the only significant within-person moderation we found and, as such, our results were not consistently different for those in longer- versus shorter-term relationships or for men versus women (see the Supplementary Online Materials for significant between-person moderations and additional exploratory analyses).

### Study 1 Limitations

Although our findings generally supported our hypotheses, they were somewhat inconsistent across different sleep facets and outcomes. In particular, results were more consistent for subjective sleep quality than sleep duration. Furthermore, sleep quality was not assessed during all daily surveys, which reduced our statistical power and may systematically bias effects. Specifically, we may be missing data from participants with some of the poorest sleep quality as partners with fewer conflicts may also be the ones reporting better overall sleep (Gordon & Chen, 2014). Given that support was not assessed every day, we also could not consistently account for the previous day’s support (which may influence sleep quality that night). Thus, we aimed to replicate our results in a similar naturalistic setting without limitations regarding when our variables of interest were assessed (i.e., on days without conflicts of interest) and how they were operationalized (i.e., sleep quality assessed with regard to fatigue). As such, we conducted a conceptual replication in a second daily diary sample to examine the robustness of our conclusions.

## Study 2: Method

### Participants

Participants were eligible to participate if they were at least 18 years or older and had been in a romantic relationship for at least three months (*M* = 2.84 years, SD = 4.41). Our sample consisted of 100 couples (*N* = 200; 87 heterosexual, 1 gay, 9 lesbian, 3 non-binary) from the United Kingdom who were recruited through social media posts, advertisements in local magazines, and at local wedding fairs. We selected our sample size a priori based on available resources and APIMPowerR analyses (https://robert-a-ackerman.shinyapps.io/APIMPowerRdis/ ) indicating that 100 couples would provide 84% power for small-to-medium cross-sectional effects. Similar to Study 1, however, our sample size again likely left us slightly underpowered to detect between-person effects, and thus, we focus on within-person effects.

Participants in our sample ranged in age from 18 to 64 years old (*M* = 24.15, *SD* = 6.61; 52.5% women, 45.5% men, 2% non-binary). Most participants (85.5%) were casually or exclusively dating their partner, while 6.5% were married, 5% were engaged, 1.5% were common law, and 1.5% were in a civil partnership. The sample was predominantly White and included participants with the following self-reported backgrounds: 85.5% White, 3% Hispanic/Latinx, 1.5% East Asian, 2.5% South Asian, 2.5% Southeast Asian, 3% bi- or multi-ethnic, and 2% other. Prior to analyses, we removed two dyads who consistently reported sleep values that were impossible (e.g., sleeping 30 h in one night) or highly unlikely (e.g., sleeping zero hours three nights in a row).

### Procedure

Couples completed a multi-part study^2^ that consisted of an in-lab session and a 14-day daily diary. After completing a 2-h in-lab session, which included demographic assessments, each participant received £16.00 as monetary compensation and was scheduled to begin the daily diary surveys the following day. Both partners were independently emailed a questionnaire at 4:00 pm each evening and given until 12:00 am to answer questions that assessed their experiences and behavior that day, including their subjective sleep quality and duration the previous night, negative affect, perspective-taking, perceptions of their partner’s perspective-taking, provision of partner support, and perceptions of their partner’s provision of support. Although participants completed all survey questions at the same time, they were nonetheless asked to reflect on different parts of their day when answering questions about our variables of interest (e.g., they were asked about their negative affect over the past 24 h, but were specifically asked to reflect on their sleep quality from the night before). At the end of the 2 weeks, participants were compensated up to £28.00 (prorated based on the number of surveys completed) for the daily diary.

### Daily Measures

#### Sleep Quality

Each day, participants rated their subjective sleep quality from the night before (“How would you rate your sleep quality last night?”) on a 4-point scale (1 = *very bad*, 4 = *very good*; *M* = 3.01, *SD* = 0.81) and sleep duration (“Please indicate how many hours of actual sleep you got last night”) using an open-ended response option (*M* = 7.56 *SD* = 1.51). As in Study 1, we again deviated from our preregistration and examined the separate effects of subjective sleep quality and sleep duration on support provision (see the Supplementary Online Materials for analyses utilizing a more holistic sleep composite).

#### Perspective-Taking

Each day, participants also rated their own perspective-taking with three items (“In the past 24 h, I really tried to understand my partner’s thoughts and feelings,” “In the past 24 h, I tried to understand my partner better by imagining how things look from their perspective,” and the reverse-scored item, “In the past 24 h, I sometimes found it difficult to see things from my partner’s point of view”) and their perceptions of their partner’s perspective-taking with three items (“In the past 24 h, my partner really tried to understand my thoughts and feelings,” “In the past 24 h, my partner tried to understand me better by imagining how things look from my perspective,” and the reverse-scored item, “In the past 24 h, my partner sometimes found it difficult to see things from my point of view”) using a 5-point scale (1 = *not at all true,* 5 = *extremely true*).

However, the *R*_*C*_ (i.e., within-person reliability) for self-reported (*R*_*C*_ = 0.30) and partner-perceived (*R*_*C*_ = 0.48) perspective-taking did not meet our preregistered cut-off of 0.70 when the reverse-scored items were included and, as such, these items were excluded from analyses. Moreover, given that the within-person correlations between the two self-reported and partner-perceived perspective-taking items were still only 0.30 (*p* < 0.001) and 0.36 (*p* < 0.001), respectively, we conducted separate analyses with the individual items rather than creating perspective-taking composites. Results using the perspective-taking items tapping into thoughts and feelings (i.e., “In the past 24 h, I really tried to understand my partner’s thoughts and feelings”; *M* = 3.79, *SD* = 1.10, “In the past 24 h, my partner really tried to understand my thoughts and feelings”; *M* = 3.67, *SD* = 1.19) are presented in the main text as this operationalization of perspective-taking most closely matches the items used in Study 1 (see the Supplementary Online Materials for analyses conducted with the additional perspective-taking items).

#### Negative Affect

Similar to Study 1, participants rated the degree to which they felt three negative emotions over the course of the day (“Indicate the extent to which you felt ‘upset,’ ‘sad,’ and ‘hostile’ in the past 24 h”) on a 5-point scale (1 = *not at all*, 5 = *a great deal*), which we then averaged to create a negative affect composite (*M* = 1.55, *SD* = 0.73, *R*_*C*_ = 0.73).

#### Partner Support Provision

Each day, using the stem “In the past 24 h…” participants rated the support they provided to their partner with six items, including: “I gave my partner a compliment or encouragement,” “I said or did something that made my partner feel loved,” “I listened to or comforted my partner,” “I was open and receptive to things my partner asked of me,” “I showed an interest in the events of my partner’s day,” and “I helped my partner out with something important” using a 5-point scale (1 = *not at all*, 5 = *very much*). We subsequently averaged across these six items to create a self-reported support provision composite (*M* = 3.81, *SD* = 0.88, *R*_*C*_ = 0.78). These items were adapted from established support and positive interaction scales (Finkenauer et al., 2010; Gable et al., 2003; Neff & Karney, 2005; Reis et al., 2014).

#### Perceived Partner Support Provision

Each day, participants also completed the same six support items with regard to the support they perceived from their partner (e.g., “In the past 24 h my partner helped me out with something important”) on the same 5-point scale. We then averaged across these six items to create a partner-perceived support provision composite (*M* = 3.81, *SD* = 0.95, *R*_*C* =_ 0.82).

## Study 2: Results

### Analysis Overview

In accordance with our preregistration, we conducted APIM and mediation analyses comparable to those in Study 1. Within-person correlations among the variables—which account for nesting—are presented in Table 4.

**Table 4:** Within-person correlations among diary variables (Study 2)

	1	2	3	4	5	6	7	8	9	10	11
1. Subjective sleep quality	—										
2. Sleep duration	.34**	—									
3. Provided support (self-reported)	.04*	− .02	—								
4. Received support (self-reported)	.04*	− .03	.60**	—							
5. Provided support (partner-reported)	− .01	.01	.28**	.38**	—						
6. Received support (partner-reported)	.06**	.03	.38**	.33**	.60**	—					
7. Negative affect (self-reported)	− .12**	− .02	− .20**	− .15**	− .06**	− .14**	—				
8. PT (self-reported)	.00	.01	.51**	.40**	.14**	.21**	− .06**	—			
9. Perceived PT (self-reported)	.02	− .02	.43**	.64**	.28**	.24**	− .08**	.48**	—		
10. PT (partner-reported)	− .04	− .01	.14**	.21**	.51**	.41**	.06**	.13**	.21**	—	
11. Perceived PT (partner-reported)	.02	.02	.28**	.24**	.43**	.64**	− .07**	.21**	.20**	.48**	—

*PT*, perspective-taking. Within-person correlations were calculated in R version 4.1.1 (R Core Team, 2020) with the rmcorr (Bakdash & Marusich, 2021) package. *p < . 05, **p < .01

### Main Effects of Impaired Sleep on Support Provision

#### Subjective Sleep Quality

Within-person subjective sleep quality results are shown in Table 5, and between-person effects are shown in the Supplementary Online Materials. Largely in line with our predictions and Study 1 results, participants’ daily subjective sleep quality (i.e., their subjective sleep quality on a given night compared to their own average subjective sleep quality) was associated with their own support provision, but not with their perceptions of the support they received from their partner. Specifically, when participants slept worse than usual, they reported providing less support to their partner but did not report receiving any less support from their partner that day. Partners additionally reported receiving less support, corroborating participant’s self-reported provision of less support, but did not report providing less support to their partners (i.e., the poor sleepers) when they slept worse than usual.

**Table 5:** Within-person effects of subjective sleep quality and sleep duration on support provision (Study 2)

Predictors	*b*	*SE*	*df*	*t*	*p*	*R*^2^
Provided support (self-reported)						
Sleep quality	0.04*	0.02	2150.47	2.32	.02	0.002
Sleep duration	0.001	0.01	2156.34	0.10	.92	—
Controlling for previous day’s self-reported provided support						
Sleep quality	0.03	0.02	1832.42	1.39	.16	—
Provided support (partner-reported)						
Sleep quality	− 0.01	0.02	2150.55	− 0.59	.55	—
Sleep duration	0.01	0.01	2156.30	1.13	.23	—
Received support (self-reported)						
Sleep quality	0.23	0.02	2115.45	1.37	.17	—
Sleep duration	− 0.01	0.01	2116.14	− 0.67	.50	—
Received support (partner-reported)						
Sleep quality	0.06**	0.02	2115.57	2.87	.004	0.004
Sleep duration	0.02^†^	0.01	2116.11	1.91	.06	0.002
Controlling for previous day’s partner-reported received support						
Sleep quality	0.06**	0.02	1827.06	3.05	.002	0.004

^✝^*p* < .10, *p < .05, **p < .01

Given that this study assessed sleep in every survey and support provision the day before may influence sleep quality that night, we also examined the same associations while controlling for support provision the previous day. As shown in Table 5, when accounting for the previous day’s support, participants no longer reported providing less support to their partner when they slept worse than usual, but their partners continued to report receiving less support from them.

#### Sleep Duration

Consistent with Study 1 results, participants’ daily sleep duration (i.e., their sleep duration on a given night compared to their own average sleep duration) was not significantly associated with any of the outcome variables (see Table 5 for within-person effect and the Supplementary Online Materials for between-person effects).

### Mediating Effects of Negative Affect and Perspective-Taking on Impaired Sleep and Support

#### Subjective Sleep Quality

Largely consistent with our predictions and Study 1 results, participants’ daily negative affect accounted for the direct association between their daily subjective sleep quality and their own support provision, as well as the direct association between participants’ daily subjective sleep quality and their partner’s perceptions of the support provision they received when also accounting (separately) for self-reported and perceived perspective-taking. These findings indicate that when people slept worse than usual the previous night, they reported experiencing greater negative affect that day and, in turn, reported providing less support to their partner and had partners who reported receiving less support from them. As in Study 1, however, all confidence intervals for within-person reported and perceived perspective-taking as a mediator in these same models contained zero and, as such, were not significant (see Table 6 for all within-person effects and the Supplementary Online Materials for all between-person effects).

**Table 6:** Within-person effects of subjective sleep quality and duration on support mediated by negative affect and perspective-taking (Study 2)

Predictors	Mediators	*a*	*b*	*ab*	*c*	*c’*	95% CI	
							Lower	Upper
Provided support (self-reported)								
Sleep quality	NA (and self-reported PT)	− 0.09***	− 0.15***	0.01	0.04*	0.01	0.01	0.02
Sleep duration	NA (and self-reported PT)	− 0.01	− 0.16***	0.002	0.001	− 0.001	− 0.001	0.01
Sleep quality	NA (and own perceived PT)	− 0.10***	− 0.17***	0.02	0.04*	0.02	0.01	0.02
Sleep duration	NA (and own perceived PT)	− 0.01	− 0.17***	0.002	0.001	− 0.004	− 0.001	0.01
Sleep quality	NA (and partner-perceived PT)	− 0.09***	− 0.18***	0.02	0.04*	0.01	0.01	0.02
Sleep duration	NA (and partner-perceived PT)	− 0.01	− 0.18***	0.002	0.001	− 0.01	0.001	0.01
Sleep quality	Self-reported PT (and NA)	0.02	0.39***	0.01	0.04*	0.01	− 0.01	0.03
Sleep duration	Self-reported PT (and NA)	0.001	0.39***	0.0004	0.001	− 0.001	− 0.01	0.01
Sleep quality	Own perceived PT (and NA)	0.02	0.28***	0.01	0.04*	0.02	− 0.01	0.02
Sleep duration	Own perceived PT (and NA)	0.01	0.28***	0.003	0.001	− 0.004	− 0.01	0.01
Sleep quality	Partner-perceived PT (and NA)	0.05^†^	0.15**	0.01	0.04*	0.01	− 0.001	0.01
Sleep duration	Partner-perceived PT (and NA)	0.04*	0.14***	0.01	0.001	− 0.01	0.001	0.01
Received support (partner-reported)								
Sleep quality	NA (and self-reported PT)	− 0.09***	− 0.12***	0.01	0.04*	0.06**	0.01	0.02
Sleep duration	NA (and self-reported PT)	− 0.01	− 0.12***	0.001	0.001	0.02^†^	− 0.001	0.004
Sleep quality	NA (and own perceived PT)	− 0.10***	− 0.13***	0.01	0.04*	0.06**	0.01	0.02
Sleep duration	NA (and own perceived PT)	− 0.01	− 0.14***	0.001	0.001	0.02^†^	− 0.001	0.005
Sleep quality	NA (and partner-perceived PT)	− 0.09***	− 0.07***	0.01	0.04*	0.06**	0.003	0.01
Sleep duration	NA (and partner-perceived PT)	− 0.01	− 0.08***	0.001	0.001	0.02^†^	− 0.001	0.003
Sleep quality	Self-reported PT (and NA)	0.02	0.10***	0.02	0.04*	0.06**	− 0.003	0.01
Sleep duration	Self-reported PT (and NA)	0.001	0.10***	0.0001	0.001	0.02^†^	− 0.003	0.003
Sleep quality	Own perceived PT (and NA)	0.02	0.21***	0.004	0.04*	0.06**	− 0.01	0.02
Sleep duration	Own perceived PT (and NA)	0.01	0.22***	0.002	0.001	0.02^†^	− 0.004	0.01
Sleep quality	Partner-perceived PT (and NA)	0.05^†^	0.50***	0.03	0.04*	0.06**	− 0.002	0.05
Sleep duration	Partner-perceived PT (and NA)	0.04*	0.50***	0.02	0.001	0.02^†^	0.0002	0.003

*NA*, negative affect. *PT*, perspective-taking. ^†^*p* < .10, *p < .05, **p < .01, ***p < .001

#### Sleep Duration

Contrary to our findings with subjective sleep quality and to our Study 1 results, all confidence intervals for within-person negative affect as a mediator contained 0 and, as such, were not significant (see Table 6 for all within-person effects and the Supplementary Online Materials for all between-person effects).

Consistent with our predictions but counter to our Study 1 results, analyses revealed a significant indirect effect of participants’ daily sleep duration on their own support provision through their partner’s perceptions of participants’ perspective-taking—but not through participants’ own self-reported or perceived perspective-taking—when also accounting for negative affect. These findings indicate that when people slept less than usual, their partner perceived them to engage in lower perspective-taking that day (even though the poor sleepers did not report the same) and, in turn, they reported providing less support to their partner.

Further in line with our predictions but counter to Study 1 results, analyses also revealed a significant indirect effect of participants’ daily sleep duration on their partner’s perceptions of the support provision they received through the partner’s perceptions of participants’ perspective-taking—but not through participants’ own self-reported or perceived perspective-taking—when also accounting for negative affect. These findings suggest that when people slept less than normal, their partner perceived them to engage in lower perspective-taking that day (even though the poor sleepers did not report the same) and, in turn, their partner reported receiving less support from them.

Demonstrating additional evidence for our proposed pathways, we again conducted reverse mediation models in which we tested if impaired sleep predicted negative affect and perspective-taking through own self-reported and partner-perceived support. In line with the notion that negative affect and perspective-taking precede support provision, only two models that included within-person self-reported and partner-perceived support as a mediator were significant (see Tables 23 and 24 in the Supplementary Online Materials for all reverse mediation confidence intervals).

### Generalizability of Findings

In a final set of analyses, following the same procedure as Study 1, we tested whether all main effect models (which included partners’ sleep) were moderated by gender or relationship length to examine the robustness of our findings. Our results revealed no significant within-person moderations by either gender or relationship length (see the Supplementary Online Materials for marginally significant moderations).

### Summary of Study 2 Results

The results of this study were fairly consistent with our hypotheses and Study 1 findings. Specifically, lower daily subjective sleep quality—but not sleep duration—was again associated with participants’ self-reported provision of less support, but not with their perceptions of receiving less support. However, this association no longer remained significant when accounting for support from the previous day, suggesting the possibility that prior day support may, in fact, be influencing sleep. Turning to partner reports, participants’ daily subjective sleep quality was not associated with their partner’s support provision but was associated with their partner’s perceptions of received support, even when accounting for the support they received the day before.

Consistent with Study 1 results, participants’ within-person negative affect again accounted for the association between their subjective sleep quality and their own support provision, as well as the association between participants’ subjective sleep quality and their partner’s perceptions of the support they received. Further in line with Study 1, but contrary to our hypotheses, perspective-taking did not consistently mediate any of the associations between impaired sleep and support provision. As such, these results suggest that negative affect may play a more important role in explaining the association between poor sleep and partner support provision.

## Discussion

People spend nearly one-third of their lives sleeping, making it crucial to determine how this overlooked biological function impacts key social processes (Gordon et al., 2017, 2021). In two naturalistic diary studies conducted in Canada and the UK, we investigated whether impaired sleep is associated with less partner support provision. Consistent with prior research, we found that participants’ poor daily subjective sleep quality—but not duration—was associated with providing less self-reported support to their partner in both studies. These findings extend previous research on older adults (e.g., Kane & Krizan, 2021) by suggesting that the effects of sleep quality on partner support are generalizable to a diverse age range of couples and highlighting the unique impact different facets of sleep may have on social processes. In particular, subjective perceptions of sleeping poorly may more negatively affect support provision than sleep duration due to small fluctuations in sleep quality being more perceptible relative to slight variations in sleep length and the need for more severe sleep deprivation to occur before negative effects of sleep duration can be observed (e.g., Parsons et al., 2021; Yoo et al., 2007).

Furthermore, participants’ poor daily subjective sleep quality was associated with their partner’s perceptions of receiving less support in Study 2 but not in Study 1, perhaps due to differences in support measurement across studies. Study 2 utilized items reflecting objectively identifiable supportive actions (e.g., encouragement, compliments), whereas Study 1 utilized a broader, more abstract measure of support (e.g., meeting a partner’s needs), which may have been more readily endorsed by participants (Haber et al., 2007). Therefore, daily sleep quality was more robustly linked to self-reported support provision than perceived support across studies. Given that only one prior study has examined self-reported and partner-perceived support simultaneously, these findings suggest that partner effects and perceived support may be affected by sleep quality in more nuanced ways, and thus may be more sensitive to differences in item measurement.

The current research also highlights at least one novel mediator that may explain sleep-support links. In line with our predictions, participants’ negative affect consistently accounted for the association between sleep quality and support provision across both studies, even in many cases in which there was not a direct link between sleep and support. Similarly, there were some indirect links between sleep duration and support provision through negative affect in Study 1. These findings are consistent with literature suggesting that sleep is strongly linked to mood which, in turn, is linked to impairments in relationship outcomes (Gordon & Chen, 2014; Ong et al., 2011). Further supporting the strong influence sleep may have on mood, perspective-taking did not consistently mediate any of the associations between impaired sleep and support provision in either study. Therefore, although poor sleep may diminish cognitive resources that are important for effective support provision and perception (e.g., Engle-Friedman & Young, 2019), it appears that negative affect may be a more important mechanism in explaining this sleep-support link.

Despite these advantages, the current research also has limitations that give rise to fruitful directions for future research. First, both studies were correlational, and as such, it is beyond the scope of our findings to address whether impaired sleep causes less reported or perceived support. Although there is more robust and experimental evidence for the link from sleep to negative affect (Franzen et al., 2008), perspective-taking (Deliens et al., 2015, 2018), and support (Yorgason et al., 2018) than the reverse, the association between subjective sleep quality and self-reported support provision (but not partner-perceived support) was no longer significant when controlling for previous day support. Thus, the link between sleep quality and support is likely bidirectional such that receiving high-quality support may also reduce anxiety and subsequently contribute to better sleep (Selcuk et al., 2017; Troxel, 2010). As a result, further experimental research is needed (e.g., using sleep-deprivation paradigms; Stepan et al., 2021).

Second, we utilized subjective measures of sleep and operationalized sleep quality slightly differently across the two studies, with our Study 1 measure limited to assessing fatigue upon awakening rather than sleep quality directly. Although self-reported sleep quality is strongly associated with numerous relationship outcomes (Pilcher et al., 1997; Strawbridge et al., 2004), future research should use more consistent sleep measures, including behavioral ones (e.g., actigraphy).

Third, although sleep has been linked to partner-perceived support in older couples over an 8-year period (Lee et al., 2017), our studies span relatively short time periods and do not examine what factors contribute to poor sleep quality ratings. As such, further studies should examine whether patterns are similar for younger couples and over larger longitudinal gaps, as well as explore individual characteristics (e.g., personality) that contribute to poor sleep quality.

Altogether, our findings extend research on the psychosocial influences of sleep and have important implications for cultivating supportive interactions among romantic partners. By contributing to our understanding of factors that influence provided and received support and mediators that may explain links between sleep and relationship functioning, future research can work towards interventions that target these key processes and may improve sleep quality, emotion regulation, or perspective-taking skills.

## Supplementary Information

Below is the link to the electronic supplementary material.

Supplementary file1 (DOCX 531 KB)

## Footnotes

1 Two weeks after completing the daily diary, participants completed a follow-up survey, which we do not analyze here.

2 Two months after completing the diary surveys, participants completed a follow-up survey, which we do not analyze here.

## Additional Information

### Availability of data and material

Data, code, materials, and preregistrations are accessible at: https://osf.io/j8kce/.

### Code availability

Code is accessible at: https://osf.io/j8kce/.
