# Supplementary material for: Daily Sleep Quality and Support in Romantic Relationships: The Role of Negative Affect and Perspective-Taking
Source: Affect Sci. 2023 Mar 3;4(2):370–84. doi: 10.1007/s42761-023-00180-7 (PMC10247628; doi:10.1007/s42761-023-00180-7)
Supplement: Supplementary file 1 — Supplementary file1 (DOCX 531 KB) [file 42761_2023_180_MOESM1_ESM.docx]

**Supplementary Online Materials (SOM)**

In the main text, we reported results for two daily diary studies. In addition to these studies, we also tested our hypotheses during a distress and change conversation in the laboratory using the same sample as Study 1. We initially only included the distress conversation, but later preregistered parallel analyses in the change conversation due to self-reported and rater-observed negative affect having more variability in this second conversation and, as such, potentially indicating a higher need for support. Details regarding the procedures for these conversations and subsequent results are presented below.

**Lab Study: Procedure**

Upon arriving at the laboratory, both partners independently completed a questionnaire that measured background information, including recent subjective sleep quality and duration. The couples then participated in a video-recorded interaction where they first took turns discussing a distressing event in their life—unrelated to their partner or relationship—that caused them suffering and continued to affect them in the present. To determine who would share their experience first, partners were randomly assigned to the role of the speaker or the listener for the distress discussions. Partners then swapped roles and went through the same process for the subsequent distress conversation. This interaction was followed by a second set of discussions where participants each spoke about an aspect they hoped to change about their partner, following the same procedure as the distress conversations.

The discussions each lasted six minutes and were adapted from a conversational structure used in past research (Fritz et al., 2003). Couples were encouraged to interact as they normally would and were informed via intercom when they were expected to speak or listen by the experimenter, who was in a separate room and not visible to participants. For the distress conversation, the partner in the speaker role was first instructed to tell their partner (i.e., the listener) about the distressing event in their life that was unrelated to their relationship. When they were ready, speakers talked about their negative experience for one minute while their partner listened. Listeners were then granted one minute to respond to what their partner (i.e., the speaker) had said. Speakers were subsequently given an additional minute to talk, followed by another minute for the listeners. Both partners were then allotted a final two minutes to speak freely with one another about the speaker’s topic. After this discussion, speakers and listeners independently completed a brief questionnaire assessing their negative affect during the conversation. Listeners additionally reported on their support provision during the discussion. Participants then completed a second distress conversation following the same procedure, in which the speakers and listeners switched roles.

Similarly, for the change conversation, the partner in the speaker role was first instructed to tell their partner about the aspect they wanted them to change, work on, or improve. Speakers then talked about this topic for one minute while their partner listened. Listeners were again given one minute to respond, after which the speaker was granted an additional minute to talk. Listeners were allotted one more minute to speak before both partners were given two minutes to talk freely. After this discussion, speakers and listeners independently completed a brief questionnaire assessing their negative affect during the conversation. Speakers additionally reported on their support provision during the discussion. Topics of change raised by the speakers included personal characteristics (e.g., anger, communication skills) and behaviors (e.g., exercise, phone use).

In total, couples engaged in seven brief discussions^[[1]](#footnote-1)^, but only conversations pertaining to

participants’ distressing experiences and desired partner changes were analyzed in the present study. Following the end of all seven in-lab discussions, each partner received $30 CAD as monetary compensation.

**Lab Study: Measures**

**Sleep Quality.** Prior to the distress and change discussions, participants rated their subjective sleep quality (“During the past month, how would you rate your overall sleep quality?”) on a 5-point scale (1 = *very bad*, 5 = *very good*) and their sleep duration (“In the last week, how many hours of sleep did you average per night?”) using an open-ended response option.^[[2]](#footnote-2)^ We then standardized and averaged these items to create a sleep quality composite (= .64, *M* = 0.00, *SD* = 0.87). Importantly, our lower obtained alpha is common for sleep composites because we are evaluating different dimensions of sleep and would not expect these distinct components to be highly related in this context (Buysse, 2014).

**Perspective-Taking.** Following the distress discussions^[[3]](#footnote-3)^, four independent coders (who were blind to study hypotheses) watched the video-recorded interactions and rated the extent to which each listener took their partner’s perspective during the conversation (“How much

perspective-taking did this person show in reaction to what their partner was saying?”) using a 5-point scale (1 = *none at all*, 5 = *an extreme amount*). We then averaged across the four coders to create a perspective-taking composite (ICC = .70, *M* = 3.28, *SD* = 0.69).

**Negative Affect.** Immediately following the distress and change discussions, listeners (during the distress conversation) and speakers (during the change conversation) rated the extent to which they felt sad, upset, down, anxious, stressed, nervous, and angry during the conversation on a 10-point scale (1 = *not at all*, 10 *= as much as I’ve ever felt*). We then averaged across these seven items to create a self-reported negative affect composite for the distress conversation (= .84, *M* = 2.92, *SD* = 1.71) and for the change conversation (= .92, *M* = 2.96, *SD* = 2.07). An additional four independent coders (who were blind to study hypotheses) also watched the video-recorded interactions and rated the extent to which each listener (during the distress conversation) and speaker (during the change conversation) expressed negative emotions towards their partner during the discussion (“How much annoyance/anger/frustration at their partner did this person express?”) using a 5-point scale (1 = *none at all*, 5 = *extremely*). We again averaged across the four coders to create an observer-rated negative affect composite for the distress conversation (ICC = .81, *M* = 1.04, *SD* = 0.12) and for the change conversation (ICC = .84, *M* = 1.66, *SD* = 0.54).

**Partner Support Provision.** Directly after the distress and change discussions, listeners (during the distress conversation) and speakers (during the change conversation) additionally rated the degree to which they were supportive of their partner during the discussion (“I was supportive of my partner to help them feel less negative emotion”) on a 7-point scale (1 = *strongly disagree*, 7 = *strongly agree*). The same four coders who rated negative affect also watched the video-recorded interactions and rated the quality of emotional support each listener (during the distress discussion) provided to their partner (“How much emotional support was this person providing?”) on a 5-point scale (1 = *none at all*, 5 = *extremely*) and the overall quality of support each listener (during the distress conversation) provided to their partner (“How would you rate the overall quality of support this person provided to their partner?”) on a 7-point scale (1 = *very poor*, 7 = *very good*).^[[4]](#footnote-4)^ We subsequently averaged across coders for both support measures to initially create an emotional support item for the distress conversation (ICC = .74, *M* = 2.86, *SD* = 0.72) and an overall support item for the distress conversation (ICC = .75, *M* = 4.97, *SD* = 0.91). We then standardized and averaged across emotional and overall support to create a partner support provision composite for the distress conversation (= .82, *M* = 0.00, *SD* = 0.92) as the correlation between these items met our preregistered minimum value for combination (*r* = .70).

**Lab Study: Analysis Overview**

We conducted two-level multilevel model analyses with participants nested within dyads to account for the non-independence in our data. Our analytic models were guided by the actor-partner interdependence model (APIM; Cook & Kenny 2005) and simultaneously included both partners’ sleep variables as predictors. The intraclass correlation coefficients (ICCs) for our dependent variables (i.e., self-reported, partner-reported, and observer-rated support provision) ranged from ICC = .76 to ICC = .90, suggesting that our outcome variables were highly nested and thereby supporting our use of multilevel modeling. With regard to model parameters, we specified model intercepts as random and slopes as fixed using restricted maximum likelihood estimation. All predictors were also grand-mean-centered prior to running analyses. We conducted main effect and moderation analyses in SPSS Version 26 and mediation analyses with the Monte Carlo Method for Assessing Mediation (MCMAM; Selig & Preacher, 2008). We estimated the indirect effect of negative affect and perspective-taking in each model using bootstrapped 95% confidence intervals based on 20,000 samples. Confidence intervals that did not include 0 were considered significant. Importantly, results using the sleep quality composite are presented in-text, while results using the individual subjective sleep quality and duration items are presented in Tables 1–4 due to the pattern of the findings being largely consistent across all three measures.

**Lab Study Results: Main Effects of Sleep Quality (Composite) on Support Provision**

We predicted that people who reported poor (vs. high) quality sleep would report providing less support to their partner during a distress and change conversation in the laboratory and be observed as providing less support to their partners during a distress conversation. Counter to our predictions, participants’ sleep quality was not associated with observer-rated support provision during the distress conversation (see Tables 1 and 2 for all main effect results). Also contrary to our predictions, participants’ sleep quality was not associated with their own self-reported support provision during the change conversation, but was associated with their own self-reported support provision during the distress conversation albeit in the unexpected direction. Specifically, people with poor quality sleep reported providing *more* support to their partner during the in-lab distress conversation. Therefore, participants who slept poorly did not provide any less support to their partners as rated by observers, but did self-report providing more support to their partners during the distress conversation.

We also predicted that people who reported poor (vs. high) quality sleep would have *partners* who would report providing less support to the poor sleepers during a distress and change conversation in the laboratory and be observed as providing less support to the poor sleepers during a distress conversation. Counter to our predictions, participants’ sleep quality was not associated with observer-rated support provision during the distress conversation. Consistent with our predictions, however, participants’ sleep quality was associated with their own self-reported support provision during the distress and change conversations, although this effect was only marginal during the change conversation. In particular, people with poor quality sleep reported providing *less* support to their partner during the in-lab distress conversation and *marginally less* support to their partner during the in-lab change conversation. Thus, participants who slept poorly did not have partners who provided any less support to them as rated by observers, but did have partners who generally reported providing less support.^[[5]](#footnote-5)^

**Lab Study Results: Mediating Effects of Negative Affect and Perspective-Taking on Sleep Quality and Support**

**Negative Affect.** We predicted that people who reported poor (vs. high) quality sleep would report providing less support to their partner during a distress and change conversation and be observed as providing less support to their partner during a distress conversation through the poor sleepers’ greater reported and observed negative affect. In line with our predictions, participants’ self-reported negative affect, but not rater-observed negative affect, accounted for the indirect association between their sleep quality and their own self-reported support provision during the change conversation (see Tables 3 and 4 for all negative affect mediation results). These findings suggest that people with poor quality sleep reported experiencing greater negative affect during the change conversation (even though raters did not observe the same) and, in turn, reported providing less support to their partner during said discussion. Counter to our predictions, however, all models that included self-reported and observed negative affect as a mediator during the distress conversation produced confidence intervals that contained 0 and, as a result, were not significant. Therefore, participants’ own self-reported and observed negative affect during the distress conversation did not mediate any of the associations between sleep quality and participants’ own self-reported or observed support provision.^[[6]](#footnote-6)^

**Perspective-Taking.** We similarly predicted that people who reported poor (vs. high) quality sleep would report and be observed as providing less support to their partners during a distress conversation through the poor sleepers’ lower observed perspective-taking. Inconsistent with our predictions, all models that included observed perspective-taking as a mediator produced confidence intervals that contained 0 and, as a result, were not significant (see Table 3 for all perspective-taking mediation results). Thus, participants’ observed perspective-taking did not mediate any of the associations between sleep quality and participants’ own self-reported or observed support provision during the distress conversation.^[[7]](#footnote-7)^

**Lab Study Results: Moderations by Gender and Relationship Length**

In a final set of analyses, we tested whether all main effect models (which included both

partners’ sleep) were moderated by participants’ gender or relationship length to examine the robustness of our findings. Our results revealed that gender moderated the association between participants’ sleep quality and their partner’s self-reported support provision during the change conversation (see Figure 1). Specifically, we found that men with poor sleep quality did not have partners who reported providing any less support to them, whereas women with poor sleep quality did have partners who reported providing less support during the change conversation. We also found that relationship length marginally moderated the association between sleep quality and participants’ own self-reported support provision during the distress conversation (see Figure 2). Among participants who had been in their relationship for a longer duration of time, sleep quality was not significantly associated with participants’ own self-reported support provision. In contrast, participants who had been in their relationship for a shorter duration of time reported providing *more* support to their partners during the distress conversation when they slept poorly. Therefore, our results were not consistently different for men versus women or those in longer- versus shorter-term relationships.

**Main Text Between-Person Effects**

In the main text, we reported within-person main effect and mediation results. Although they were not the primary focus of our studies, our analyses also allowed to us to separate between-person main effect and mediations results. As such, we present Study 1 between-person main effect and mediation results in Tables 5 and 6 and Study 2 between-person main effect and mediation results in Tables 7 and 8.

**Main Text Analyses Conducted with Holistic Sleep Measures**

In the main text, we reported main effect and mediation results when using subjective sleep quality and sleep duration as separate predictors. Consistent with our preregistration, we also conducted the same analyses in Studies 1 and 2 using a more holistic sleep composite. Details regarding how these composites were created and the results of these additional analyses are presented below.

**Study 1: Sleep Quality Composite**

In Study 1, participants rated their subjective sleep quality (“When I woke up today, I felt:_____?”) on a 4-point scale (1 = *refreshed*, 4 = *fatigued)*, sleep latency (“Last night I feel asleep in ____?”) on a 4-point scale (1 = *0-15 minutes*, 4 = *more than 60 minutes)*, level of enthusiasm (“Today, how much of a problem has it been for you to keep up enough enthusiasm to get things done?”) on a 4-point scale (1 = *no problem at all*, 4 = *a very big problem)*, tiredness (“How tired were you today?”) on a 5-point scale (0 = *not at all*, 4 = *extremel*y), number of awakenings (“I woke up ____ times during the night?”) using an open-ended response option, and sleep duration (“I slept a total of ____ hours?”) using an open-ended response option. Given that level of enthusiasm and tiredness assess the same aspect of sleep (i.e., daytime dysfunction), we first standardized and averaged across these two items to create a daytime dysfunction composite (= .67, *M* = 0.04, *SD* = 0.88). We then standardized the four remaining sleep items and averaged them with the daytime dysfunction item to create a sleep quality composite (= .59, *R_C_* = .74, *M* = 0.01, *SD* = 0.60). Importantly, our lower obtained alpha is common for sleep composites because we are evaluating different dimensions of sleep and would not anticipate these distinct components to be highly related in comprehensive assessment (Buysse, 2014).

**Study 1: Main Effects of Sleep Quality (Composite) on Support Provision**

In line with our predictions, participants’ daily sleep quality (i.e., their sleep quality on a given night compared to their own average sleep quality) was associated with their own support provision and their perceptions of the support they received from their partner, although this effect was only marginally significant (see Table 9 for all within- and between-person results). Specifically, when participants slept worse than usual, they reported providing less support to their partner and receiving marginally less support from their partner that day. Counter to our predictions, however, participants’ daily sleep quality was not associated with their partner’s support provision or their partner’s perceptions of the support provision they received. As such, when participants slept worse than normal, their partner did not report providing or receiving any less support from them (i.e., the poor sleepers). In other words, on days when participants slept worse than usual, they were less likely to report meeting their partner’s needs, but their partner was not more likely to perceive this lack of support. While participants were also marginally less likely to perceive their partner as meeting their needs, their partner was not more likely to report providing any less support.

**Study 1: Mediating Effects of Negative Affect on Sleep Quality (Composite) and Support**

Consistent with our predictions, participants’ daily negative affect accounted for the direct association between their daily sleep quality and their own support provision, as well as the indirect association between participants’ daily sleep quality and their partner’s perceptions of the support provision they received (see Table 10). These results suggest that when people slept worse than usual, they reported experiencing greater negative affect that day and, in turn, reported providing less support to their partner and had partners who reported receiving less support from them.

**Study 1: Mediating Effects of Perspective-Taking on Sleep Quality (Composite) and Support**

Contrary to our predictions, participants’ daily perspective-taking and their partners’ perceived perspective-taking did not account for the association between participants’ sleep quality and their own support provision (see Table 10). Based on these findings, we conducted additional exploratory analyses (which we preregistered in Study 2) to investigate mediations with perceived partner perspective-taking and found that participants’ daily perceptions of their partners’ perspective-taking accounted for the direct association between their sleep quality and their own support provision. This result suggests that when people slept worse than usual, they reported that their partner engaged in less perspective-taking that day and, in turn, reported providing less support to their partner. Therefore, participants’ perceived perspective-taking helped explain the link between sleep quality and support provision, but their own perspective-taking and partners’ perceptions of the poor sleeper’s perspective-taking did not.

Also counter to our predictions, participants’ daily perspective-taking and their partners’ perceived perspective-taking did not account for the association between participants’ sleep quality and their partner’s perceptions of the support provision they received either. As with self-reported support, we again conducted exploratory analyses (which we preregistered in Study 2) to investigate mediations with perceived partner perspective-taking but did not find any significant associations.

**Study 1: Mediating Effects of Negative Affect on Impaired Sleep and Support**

**Subjective Sleep Quality.** Mostly consistent with our predictions, participants’ daily negative affect accounted for the direct association between their daily subjective sleep quality and their own support provision, but not their partner’s perceptions of the support provision they received (see Table 11 for all within-person model statistics controlling for partner’s sleep and Table 12 for between-person effects). These results suggest that when people felt more fatigued than usual, they reported experiencing greater negative affect that day and, in turn, reported providing less support to their partner even though their partner did not report perceiving less support from them.

**Sleep Duration.** Participants’ daily negative affect also accounted for the indirect association between their daily sleep duration and their own support provision as well as the indirect association between participants’ daily sleep duration and their partner’s perceptions of the support provision they received (see Table 11 for all within-person model statistics controlling for partner’s sleep and Table 12 for between-person effects). These findings indicate that when people slept less than normal the previous night, they reported experiencing greater negative affect that day and, in turn, reported providing less support to their partner and had partners who reported receiving less support from them.

**Study 1: Mediating Effects of Perspective-Taking on Impaired Sleep and Support**

**Subjective Sleep Quality.**  Largely in line with our predictions, participants’ daily perspective-taking, but not their partners’ perceived perspective-taking, accounted for the direct association between participants’ subjective sleep quality and their own support provision (see Table 11 for all within-person model statistics controlling for partner’s sleep and Table 12 for between-person effects). These findings indicate that when people felt more fatigued than usual upon waking, they reported engaging in lower perspective-taking that day (even though their partner did not perceive the same) and, in turn, reported providing less support to their partner. Given these findings, we conducted additional exploratory analyses (which we preregistered in Study 2) to investigate mediations with perceived partner perspective-taking and found that participants’ daily perceptions of their partners’ perspective-taking also accounted for the direct association between their subjective sleep quality and their own support provision. This result suggests that when people felt more fatigued than usual, they reported that their partner engaged in less perspective-taking that day and, in turn, reported providing less support to their partner. Therefore, participants’ own self-reported and perceived perspective-taking both helped explain the link between sleep quality and support provision, but partners’ perceptions of the poor sleeper’s perspective-taking did not.

Further supporting most of our predictions, analyses also revealed a significant indirect effect of participants’ daily subjective sleep quality on their partner’s perceptions of the support provision they received through participants’ daily perspective-taking, but not through their partners’ perceived perspective-taking. These findings indicate that when people felt more fatigued than usual, they reported engaging in lower perspective-taking that day (even though their partner did not perceive the same) and, in turn, had partners who reported receiving less support from them. However, as with self-reported support, exploratory analyses (which we preregistered in Study 2) conducted to investigate mediations with perceived partner perspective-taking revealed that participants’ daily perceptions of their partner’s perspective-taking additionally accounted for the indirect association between their subjective sleep quality and their partner’s perceptions of the support provision they received. This result suggests that when people felt more fatigued than normal, they perceived their partner as engaging in less perspective-taking that day and, in turn, had partners who reported receiving less support from them. Thus, participants’ own self-reported and perceived perspective-taking both helped explain the link between sleep quality and partner-perceived support provision, but partners’ perceptions of the poor sleeper’s perspective-taking did not.

**Sleep Duration.** All confidence intervals for models that included within-person reported and perceived perspective-taking as a mediator contained zero and, as such, were not significant (see Table 11 for all within-person model statistics controlling for partner’s sleep and Table 12 for between-person effects).

**Study 1: Gender and Relationship Length Moderations**

In a final set of analyses, we tested whether all main effect models (which included both

partners’ sleep) were moderated by participants’ gender or relationship length to examine the robustness of our findings. Our results revealed that gender moderated the association among between-person subjective sleep quality and participants’ self-reported support provision (see Figure 3). Specifically, we found that men with poorer subjective sleep quality as compared to other participants across the diary did not report providing any less support to their partners, whereas women with poorer subjective sleep quality as compared to other participants across the diary did report providing less support to their partners. We also found that gender moderated the association among between-person subjective sleep quality and participants’ perceptions of their partner’s support provision (see Figure 4). In this case, we again found that men with poorer sleep quality as compared to other participants across the diary did not report receiving any less support from their partners, whereas women with poorer sleep quality as compared to other participants across the diary did report receiving less support from their partners. Therefore, having poor average subjective sleep quality across the diary appeared to have a more negative impact on women’s provision of support to their partners and perceptions of support from their partners than it did on men’s provision and perceptions of support.

Similarly, our results additionally revealed that gender moderated the association among between-person sleep duration and participants’ self-reported support provision (see Figure 5). In particular, we found that men with a shorter sleep duration as compared to other participants across the diary reported providing *more* support to their partners, whereas women with a shorter sleep duration as compared to other participants across the diary reported providing *less* support to their partners. We also found that gender moderated the association among between-person sleep duration and participants’ perceptions of their partner’s support provision (see Figure 6). In this case, we again found that men with a shorter sleep duration as compared to other participants across the diary reported receiving *more* support from their partners, whereas women with a shorter sleep duration as compared to other participants across the diary reported receiving *less* support from their partners. Thus, having poor average sleep duration across the diary appeared to have a negative impact and on women’s provision of support to their partners and perceptions of support from their partners and a positive impact on men’s provision and perceptions of support.

Finally, we found that relationship length marginally moderated the link among between-person subjective sleep quality and the partners of participants’ self-reported support provision (see Figure 7). Among participants who had been in their relationship for a longer duration of time, having poorer sleep quality as compared to other participants across the diary was not significantly associated with the partners of participants’ self-reported support. In contrast, participants who had been in their relationship for a shorter duration of time had partners who reported providing marginally less support to the poor sleepers when they had poorer sleep quality as compared to other participants across the diary. Analyses also revealed that relationship length marginally moderated the link among participants’ within-person subjective sleep quality and their perceptions of received support (see Figure 8). Among participants who had been in their relationship for a shorter duration of time, having poor daily subjective sleep quality was not significantly associated with their perceptions of received support. In contrast, participants who had been in their relationship for a longer duration of time reported receiving less support from their partners when they slept worse than usual. Therefore, our results were not consistently different for those in longer- versus shorter-term relationships.

**Study 2: Sleep Quality Composite**

In Study 2, participants rated their subjective sleep quality (“How would you rate your sleep quality last night?”) on a 4-point scale (1 = *very bad*, 4 = *very good*) and sleep duration (“Please indicate how many hours of actual sleep you got last night”) using an open-ended response option. We then standardized and averaged across these two items to create a sleep quality composite (= .52, *M* = 0.00, *SD* = 0.82).

**Study 2: Main Effects of Sleep Quality (Composite) on Support Provision**

Counter to our predictions and Study 1 results, participants’ daily sleep quality was not associated with their own support provision or their perceptions of the support they received from their partner (see Table 13 for all within- and between-person results). As such, when participants slept worse than usual, they did not report providing or receiving any less support from their partner. Partially in line with our predictions, however, participants’ daily sleep quality was associated with their partner’s perceptions of the support provision they received but was not associated with their partner’s self-reported support provision. Specifically, when participants slept worse than normal, their partner reported receiving less support from them that day, but their partner did not report providing any less support to them. In other words, on days when participants slept worse than usual, their partner was less likely to report receiving support from them, but they (i.e., the poor sleepers) were not more likely to report providing less support.

**Study 2: Mediating Effects of Negative Affect (Composite) on Sleep Quality and Support**

Consistent with our predictions and Study 1 results, participants’ daily negative affect accounted for the indirect association between their daily sleep quality and their own support provision, as well as the direct association between participants’ sleep quality and their partner’s perceptions of the support provision they received (see Table 14). These results suggest that when people slept worse than usual, they reported experiencing greater negative affect that day and, in turn, reported providing less support to their partner and had partners who reported receiving less support from them.

**Study 2: Mediating Effects of Perspective-Taking (Composite) on Sleep Quality and Support**

In line with our predictions but contrary to our Study 1 results, partners’ daily perceptions of participants’ perspective-taking, but not participants’ own self-reported or perceived perspective-taking, accounted for the indirect association between sleep quality and participants’ own support provision (see Table 14). These findings indicate that when people slept worse than usual, their partner perceived them to engage in lower perspective-taking that day (even though the poor sleepers did not report the same) and, in turn, they reported providing less support to their partner. Therefore, partners' daily perceptions of participants’ perspective-taking helped explain the link between sleep quality and support provision, but participants’ own self-reported and perceived perspective-taking did not.

Further in line with our predictions but counter to our Study 1 results, partners’ daily perceptions of participants’ perspective-taking, but not participants’ own self-reported or perceived perspective-taking, also accounted for the direct association between sleep quality and partner’s perceptions of the support provision they received. These findings suggest that when people slept worse than usual, their partner perceived them to engage in lower perspective-taking that day (even though the poor sleepers did not report the same) and, in turn, their partner reported receiving less support from them. Thus, partners' daily perceptions of participants’ perspective-taking helped explain the link between sleep quality and partner’s perceptions of the support provision they received, but participants’ own self-reported and perceived perspective-taking did not.

**Study 2: Mediating Effects of Negative Affect on Impaired Sleep and Support**

**Subjective Sleep Quality.** Largely consistent with our predictions and Study 1 results, participants’ daily negative affect accounted for the direct association between their daily subjective sleep quality and their own support provision as well as the direct association between participants’ subjective sleep quality and their partner’s perceptions of the support provision they received (see Table 15 for all within-person model statistics controlling for partner’s sleep and Table 16 for between-person effects). These results suggest that when people slept worse than usual, they reported experiencing greater negative affect that day and, in turn, reported providing less support to their partner and had partners who reported receiving less support from them.

**Sleep Duration.** Contrary to our findings with subjective sleep quality and to our Study 1 results, all confidence intervals for models that included within-person negative affect as a mediator contained 0 and, as such, were not significant (see Table 15 for all within-person model statistics controlling for partner’s sleep and Table 16 for between-person effects).

**Study 2: Mediating Effects of Perspective-Taking on Impaired Sleep and Support**

**Subjective Sleep Quality.** Based on the findings from our exploratory perspective-taking analyses in Study 1, we preregistered predictions that perceived partner perspective-taking would mediate the relationship between participants’ sleep quality and self-reported and partner-perceived support provision in addition to testing our original hypotheses

Consistent with our predictions but contrary to our Study 1 results, partners' daily perceptions of participants’ perspective-taking, but not participants’ own self-reported or perceived perspective-taking, accounted for the direct association between subjective sleep quality and participants’ own support provision (see Table 15 for all within-person model statistics controlling for partner’s sleep and Table 16 for between-person effects). These findings indicate that when people slept worse than usual, their partner perceived them to engage in lower perspective-taking that day (even though the poor sleepers did not report the same) and, in turn, they reported providing less support to their partner. Therefore, partners' daily perceptions of participants’ perspective-taking helped explain the link between sleep quality and support provision, but participant’s own self-reported and perceived perspective-taking did not.

Further in line with our predictions but counter to our Study 1 results, partners daily perceptions of participants’ perspective-taking, but not participants’ own self-reported or perceived perspective-taking, also accounted for the direct association between subjective sleep and partner’s perceptions of the support provision they received. These findings suggest that when people slept worse than usual, their partner perceived them to engage in lower perspective-taking that day (even though the poor sleepers did not report the same) and, in turn, their partner reported receiving less support from them. Thus, partners' daily perceptions of participants’ perspective-taking helped explain the link between sleep quality and partner’s perceptions of the support provision they received, but participant’s own self-reported and perceived perspective-taking did not.

**Sleep Duration.** Consistent with our predictions but counter to our Study 1 results, analyses revealed a significant indirect effect of participants’ daily sleep duration on their own support provision through their partner’s perceptions of participants’ perspective-taking, but not through participants’ own self-reported or perceived perspective-taking (see Table 15 for all within-person model statistics controlling for partner’s sleep and Table 16 for between-person effects). These findings indicate that when people slept less than usual, their partner perceived them to engage in lower perspective-taking that day (even though the poor sleepers did not report the same) and, in turn, they reported providing less support *to their partner*. Therefore, partners’ daily perceptions of participants’ perspective-taking helped indirectly explain the link between sleep duration and support provision, but participant’s own self-reported and perceived perspective-taking did not.

Further in line with our predictions but counter to Study 1 results, analyses also revealed a significant indirect effect of participants’ daily sleep duration on their partner’s perceptions of the support provision they received through the partner’s perceptions of participants’ perspective-taking, but not through participants’ own self-reported or perceived perspective-taking. These findings suggest that when people slept less than normal, their partner perceived them to engage in lower perspective-taking that day (even though the poor sleepers did not report the same) and, in turn, their partner reported receiving less support *from them*. Thus, partners’ daily perceptions of participants’ perspective-taking helped explain the link between sleep duration and partner’s perceptions of the support provision they received, but participant’s own self-reported and perceived perspective-taking did not.

**Additional Perspective-Taking Mediation Analyses**

In the main text, we reported perspective-taking mediation results for Study 2 when using the items tapping into participants’ thoughts and feelings (i.e., “In the past 24 hours, I really tried to understand my partner’s thoughts and feelings,” “In the past 24 hours, my partner really tried to understand my thoughts and feelings”), which were most conceptually similar to how perspective-taking was operationalized in Study 1. We also conducted the same analyses using the second set of perspective-taking items (i.e., “In the past 24 hours, I tried to understand my partner better by imagining how things look from their perspective,” “In the past 24 hours, my partner tried to understand me better by imagining how things look from my perspective”) and reverse-scored perspective-taking items (i.e., “In the past 24 hours, I sometimes found it difficult to see things from my partner’s point of view,” “In the past 24 hours, my partner sometimes found it difficult to see things from my point of view”). Mediation results using these additional perspective-taking items are presented below.

**Study 2: Mediating Effects of Perspective-Taking (Item Two) on Impaired Sleep and Support**

**Subjective Sleep Quality.** All models that included within-person reported and perceived perspective-taking as a mediator contained 0 and, as such, were not significant (see Tables 17 and 18).

**Sleep Duration.** Consistent with our subjective sleep quality results, all models that included within-person reported and perceived perspective-taking as a mediator contained 0 and, as such, were not significant (see Tables 17 and 18).

**Study 2: Mediating Effects of Perspective-Taking (Reverse-Scored Item) on Impaired Sleep and Support**

**Subjective Sleep Quality.** Participants’ daily perceptions of their partner’s perspective-taking and their partners’ daily perceptions of their (i.e., the poor sleepers) perspective-taking, but not participants’ own self-reported perspective-taking, accounted for the association between subjective sleep quality and participants’ own support provision (see Tables 19 and 20). These findings indicate that when people slept worse than usual, they perceived their partner to engage in less perspective-taking and their partner perceived them to engage in lower perspective-taking that day (even though the poor sleepers did not report the same) and, in turn, they reported providing less support to their partner. Therefore, participants’ daily perceptions of their partner’s perspective-taking and their partners' daily perceptions of their (i.e., the poor sleepers) perspective-taking helped explain the link between sleep quality and support provision, but participants’ own self-reported perspective-taking did not.

Similarly, participants’ daily perceptions of their partner’s perspective-taking and their partners’ daily perceptions of their (i.e., the poor sleepers) perspective-taking, but not participants’ own self-reported perspective-taking, accounted for the association between subjective sleep quality and partner’s perceptions of the support provision they received. These findings suggest that when people slept worse than usual, they perceived their partner to engage in less perspective-taking and their partner perceived them to engage in lower perspective-taking that day (even though the poor sleepers did not report the same) and, in turn, their partner reported receiving less support from them. Thus, participants’ daily perceptions of their partner’s perspective-taking and their partners' daily perceptions of their (i.e., the poor sleepers) perspective-taking helped explain the link between sleep quality and partner’s perceptions of the support provision they received, but participants’ own self-reported perspective-taking did not.

**Sleep Duration.** All models that include within-person reported and perceived perspective-taking as a mediator contained 0 and, as such, were not significant (see Tables 19 and 20).

**Study 2: Gender and Relationship Length Moderations**

As with Study 1, in a final set of analyses, we again tested whether all main effect models (which included both partners’ sleep) were moderated by participants’ gender or relationship length to examine the robustness of our findings. Our results revealed that relationship length marginally moderated the association among participants’ within-person sleep duration and their partner’s perceptions of the support provision they received (see Figure 9). Among participants who had been in their relationship for a shorter duration of time, sleeping less than usual on a given day was not significantly associated with their partner’s perceptions of received support. In contrast, participants who had been in their relationship for a longer duration of time had partners who reported receiving less support from the impaired sleepers when they slept less than they usually did on a given day. This was the only marginally significant moderation we found and, as such, our results were not consistently different for those in longer- versus shorter-term relationships or for men versus women.

**Mediation Models: Analytic Approach**

The mediation models presented in the main text and here in the Supplementary Online

Materials were all run using the Monte Carlo Method for Assessing Mediation (MCMAM). Initially, we preregistered a dual-pronged approach towards our mediation models and noted that we would also use the SPSS macro MLMED to test the same mediations as two-level multilevel models in secondary analyses. However, given that we did not model random slopes, we do not need to account for the covariance between the *a* and *b* paths in our mediation models changing as a result of random slopes. As such, we do not report any mediation results run using MLMED and only report our MCMAM results both here and in the main text.

**Exploratory Moderations**

In addition to exploring moderations by gender and relationship length, we also assessed communal strength in Study 1 (including the Lab Study) and conducted exploratory moderations by communal strength given that the extent to which participants aim to meet their partner’s needs and desires may also shape support outcomes. To measure communal strength, participants answered 10 self-reported communal strength items from Lemay and Neal (2013) in the baseline questionnaire using a 7-point scale (1 = *strongly disagree*, 7 *= strongly agree*). Sample items included: “Helping my partner is a high priority for me” and “Thinking about my partner’s needs is easy for me.” All 10 items were subsequently averaged to create a communal strength composite (= .88, *M* = 6.49, *SD* = 0.54). The results of these exploratory communal strength moderations are presented below.

**Lab Study: Exploratory Communal Strength Moderations**

We conducted a set of exploratory moderation analyses to examine possible interactions between sleep quality (i.e., the composite combining subjective sleep quality and duration) and communal strength (assessed at baseline) on participants’ own self-reported and observer-rated support provision in the laboratory. We specifically examined if communal strength moderated the association between sleep quality and participants’ own self-reported support provision and the association between sleep quality and participants’ observer-rated support provision during the distress and change conversations.

Analyses revealed that communal strength marginally moderated the association between sleep quality and participants’ own self-reported support provision during the change conversation (see Figure 10). Among participants who had high levels of communal strength, sleep quality was not significantly associated with participants’ own self-reported support provision. In contrast, participants who had low levels of communal strength reported providing less support to their partners when they slept poorly. We also found that communal strength moderated the association between participants’ sleep quality and their partner’s self-reported support provision during the change conversation (see Figure 11). Among participants who had low levels of communal strength, sleep quality was not significantly associated with their partner’s self-reported support provision. Conversely, participants who had high levels of communal strength had partners who reported providing less support to them when they slept poorly. No significant communal strength moderations were found for the distress conversation.

**Study 1: Exploratory Communal Strength Moderations**

We also conducted a set of exploratory moderation analyses to examine possible interactions between impaired sleep (i.e., subjective sleep quality, sleep duration) and communal strength (assessed at baseline) on participants’ own support provision and their partner’s perceptions of support provision in the daily diary. We specifically examined if communal strength moderated the association between impaired sleep and participants’ own self-reported support provision in everyday life and the association between impaired sleep and partners’ perceptions of the support provision they received in day-to-day life. These analyses did not reveal any significant interactions.

**References**

Buysse, D. J. (2014). Sleep health: Can we define it? Does it matter? *Sleep, 37*(1), 9–17. https://doi.org/10.5665/sleep.3298

Cook, W., & Kenny, D. (2005). The Actor–Partner Interdependence Model: A model of bidirectional effects in developmental studies. *International Journal of Behavioral Development*, *29*(2), 101–109. https://doi.org/10.1080/01650250444000405

Fritz, H., Nagurney, A., & Helgeson, V. (2003). Social interactions and cardiovascular reactivity during problem disclosure among friends. *Personality and Social Psychology Bulletin*, *29*(6), 713–725. https://doi.org/10.1177/0146167203029006004

Lemay, E. P., & Neal, A. M. (2013). The wishful memory of interpersonal responsiveness. Journal of Personality and Social Psychology, 104(4), 653–672. [https://doi.org/10.1037/a0030422](https://doi.apa.org/doi/10.1037/a0030422)

Selig, J. P., & Preacher, K. J. (2008). Monte Carlo method for assessing mediation: An interactive tool for creating confidence intervals for indirect effects [Computer software]. Available from [http://quantpsy.org/](http://quantpsy.org).

**Table 1**

*The Effects of Impaired Sleep on Support Provision (Lab Study—Distress Conversation)*

| Predictors | *b* | *SE* | *df* | *t* | *p* | R^2^ | *b* | *SE* | *df* | *t* | *p* | R^2^ |
| --- | --- | --- | --- | --- | --- | --- | --- | --- | --- | --- | --- | --- |

**Provided Support (Self-Reported)**

**————————————————————————————————————————————————————––––––**

Self-Reported Support Observer-Rated Support

| SQ (Composite) | -0.18* | 0.09 | 199.35 | -2.00 | .047 | 0.01 | 0.07 | 0.08 | 193.00 | 0.93 | .35 | — |
| --- | --- | --- | --- | --- | --- | --- | --- | --- | --- | --- | --- | --- |
| SQ (Single Item) | -0.07 | 0.09 | 190.72 | -0.79 | .43 | — | 0.08 | 0.08 | 174.86 | 0.99 | .32 | — |
| SD (Single Item) | -0.18* | 0.08 | 191.00 | -2.45 | .014 | 0.02 | 0.04 | 0.06 | 187.55 | 0.73 | .47 | — |

**Provided Support (Partner-Reported)**

**————————————————————————————————————————————————————––––––**

Self-Reported Support Observer-Rated Support

| SQ (Composite) | 0.19* | 0.09 | 199.42 | 2.16 | .03 | 0.02 | -0.02 | 0.08 | 193.00 | -0.21 | .83 | — |
| --- | --- | --- | --- | --- | --- | --- | --- | --- | --- | --- | --- | --- |
| SQ (Single Item) | 0.17^†^ | 0.09 | 190.70 | 1.83 | .07 | 0.01 | 0.01 | 0.08 | 174.86 | 0.16 | .88 | — |
| SD (Single Item) | 0.10 | 0.07 | 190.93 | 1.46 | .15 | — | -0.04 | 0.06 | 187.59 | -0.59 | .56 | — |

*Note*. SQ = Sleep Quality, SD = Sleep Duration. ^†^*p* < .10, **p* < .05

**Table 2**

*The Effects of Impaired Sleep on Support Provision (Lab Study—Change Conversation)*

| Predictors | *b* | *SE* | *df* | *t* | *p* | R^2^ |
| --- | --- | --- | --- | --- | --- | --- |

**Provided Support (Self-Reported)**

**————————————————————————————————————————————————**

Self-Reported Support

| SQ (Composite) | 0.10 | 0.13 | 196.28 | 0.77 | .44 | — |
| --- | --- | --- | --- | --- | --- | --- |
|  |  |  |  |  |  |  |
| SQ (Single Item) | 0.24^†^ | 0.13 | 186.52 | 1.89 | .06 | 0.01 |
|  |  |  |  |  |  |  |
| SD (Single Item) | 0.01 | 0.10 | 189.73 | 0.09 | .93 | — |
|  |  |  |  |  |  |  |

**Provided Support (Partner-Reported)**

**————————————————————————————————————————————————**

Self-Reported Support

| SQ (Composite) | 0.23^†^ | 0.12 | 196.35 | 1.83 | .07 | 0.01 |
| --- | --- | --- | --- | --- | --- | --- |
|  |  |  |  |  |  |  |
| SQ (Single Item) | 0.23^†^ | 0.13 | 186.41 | 1.83 | .07 | 0.01 |
|  |  |  |  |  |  |  |
| SD (Single Item) | 0.13 | 0.10 | 186.77 | 1.27 | .21 | — |
|  |  |  |  |  |  |  |

*Note*. ^†^*p* < .10

**Table 3**

*The Effects of Impaired Sleep on Support Mediated by Negative Affect and Perspective-Taking (Lab Study—Distress Conversation)*

| Predictors | Mediators | *a* | *b* | *ab* | *c* | *c’* | 95% CI  Lower Upper | | |
| --- | --- | --- | --- | --- | --- | --- | --- | --- | --- |
| **Provided Support (Self-Reported)** | | | | | | | | |  |
| SQ (Composite) | Negative Affect (SR) | -0.25^†^ | 0.01 | -0.002 | -0.18* | -0.18^†^ | -0.03 | 0.02 | |
| SQ (Composite) | Negative Affect (RO) | 0.001 | -1.62* | -0.002 | -0.18* | -0.18^†^ | -0.03 | 0.03 | |
| SQ (Composite) | Perspective-Taking (RO) | 0.06 | 0.23^†^ | 0.01 | -0.18* | -0.19^†^ | -0.01 | 0.06 | |
| SQ (Single Item) | Negative Affect (SR) | -0.33* | 0.01 | -0.003 | -0.07 | -0.07 | -0.04 | 0.03 | |
| SQ (Single Item) | Negative Affect (RO) | 0.004 | -1.58* | -0.006 | -0.07 | -0.06 | -0.04 | 0.03 | |
| SQ (Single Item) | Perspective-Taking (RO) | 0.08 | 0.22^†^ | 0.02 | -0.07 | -0.05 | -0.01 | 0.06 | |
| SD (Single Item) | Negative Affect (SR) | -0.05 | 0.02 | -0.001 | -0.18* | -0.18* | -0.01 | 0.01 | |
| SD (Single Item) | Negative Affect (RO) | -0.004 | -1.70* | -0.007 | -0.18* | -0.18* | -0.02 | 0.04 | |
|  |  |  |  |  |  |  |  |  | |
| SD (Single Item) | Perspective-Taking (RO) | 0.05 | 0.26* | 0.01 | -0.18* | -0.19* | -0.01 | 0.05 | |
| **Provided Support (Rater-Observed)** | | | | | | | | |  |
| SQ (Composite) | Negative Affect (SR) | -0.25^†^ | 0.02 | -0.01 | 0.07 | 0.05 | -0.03 | 0.02 | |
| SQ (Composite) | Negative Affect (RO) | 0.001 | -3.73*** | -0.004 | 0.07 | 0.07 | -0.07 | 0.06 | |
| SQ (Composite) | Perspective-Taking (RO) | 0.06 | 0.78*** | 0.05 | 0.07 | -0.02 | -0.04 | 0.14 | |
| SQ (Single Item) | Negative Affect (SR) | -0.33* | -0.02 | 0.01 | 0.08 | 0.05 | -0.04 | 0.02 | |
| SQ (Single Item) | Negative Affect (RO) | 0.004 | -3.73*** | -0.01 | 0.08 | 0.10 | -0.09 | 0.06 | |
| SQ (Single Item) | Perspective-Taking (RO) | 0.08 | 0.79*** | 0.06 | 0.08 | -0.01 | -0.03 | 0.17 | |
| SD (Single Item) | Negative Affect (SR) | -0.05 | 0.01 | -0.001 | 0.04 | 0.03 | -0.01 | 0.01 | |
| SD (Single Item) | Negative Affect (RO) | -0.004 | -3.71*** | 0.01 | 0.04 | 0.02 | -0.03 | 0.07 | |
| SD (Single Item) | Perspective-Taking (RO) | 0.05 | 0.77*** | 0.04 | 0.04 | -0.02 | -0.03 | 0.11 | |

*Note*. SQ = Sleep Quality, SD = Sleep Duration, SR = Self-Reported, RO = Rater-Observed. ^†^*p* < .10, **p* < .05, ****p* < .001

**p* < 0.05

**Table 4**

*The Effects of Impaired Sleep on Support Mediated by Negative Affect (Lab Study—Change Conversation)*

| Predictors | Mediators | *a* | *b* | *ab* | *c* | *c’* | 95% CI  Lower Upper | | | |
| --- | --- | --- | --- | --- | --- | --- | --- | --- | --- | --- |
| **Provided Support (Self-Reported)** | | | | | | | | | |  |
| SQ (Composite) | Negative Affect (SR) | -0.40^*^ | -0.15** | 0.06 | 0.10 | 0.05 | 0.01 | | 0.14 | |
| SQ (Composite) | Negative Affect (RO) | -0.005 | -0.84*** | 0.004 | 0.10 | 0.10 | -0.07 | | 0.08 | |
| SQ (Single Item) | Negative Affect (SR) | -0.40* | -0.14* | 0.06 | 0.24^†^ | 0.19 | 0.004 | | 0.13 | |
| SQ (Single Item) | Negative Affect (RO) | -0.01 | -0.76*** | 0.01 | 0.24^†^ | 0.23^†^ | -0.06 | | 0.08 | |
| SD (Single Item) | Negative Affect (SR) | -0.27* | -0.16** | 0.04 | 0.01 | -0.02 | 0.001 | | 0.10 | |
|  |  |  |  |  |  |  | |  |  |  |
| SD (Single Item) | Negative Affect (RO) | -0.01 | -0.85*** | 0.01 | 0.01 | 0.01 | -0.06 | | 0.07 | |

*Note*. SQ = Sleep Quality, SD = Sleep Duration, SR = Self-Reported, RO = Rater-Observed. ^†^*p* < .10, **p* < .05, ***p* < .01, ****p* < .001

**p* < 0.05

**Table 5**

*Between-Person Effects of Subjective Sleep Quality and Sleep Duration on Support Provision (Study 1)*

| Predictors | *b* | *SE* | *df* | *t* | *p* | R^2^ |
| --- | --- | --- | --- | --- | --- | --- |

**Provided Support (Self-Reported)**

**————————————————————————————————————————————————**

| Sleep Quality | 0.50** | 0.14 | 203.04 | 3.62 | .001 | 0.02 |
| --- | --- | --- | --- | --- | --- | --- |
| Sleep Duration | -0.03 | 0.10 | 189.60 | -0.30 | .77 | — |

**Provided Support (Partner-Reported)**

**————————————————————————————————————————————————**

| Sleep Quality | -0.10 | 0.21 | 190.54 | -0.46 | .65 | — | |  |
| --- | --- | --- | --- | --- | --- | --- | --- | --- |
| Sleep Duration | 0.03 | 0.14 | 202.99 | 0.17 | .87 | | — | |

**Received Support (Self-Reported)**

**————————————————————————————————————————————————**

| Sleep Quality  Sleep Duration | 0.51**  -0.01 | 0.14  0.10 | 201.58  181.97 | 3.71  -0.06 | .001  .95 | 0.02  — |
| --- | --- | --- | --- | --- | --- | --- |

**Received Support (Partner-Reported)**

**————————————————————————————————————————————————**

| Sleep Quality | 0.08 | 0.14 | 201.15 | 0.58 | .56 | — |
| --- | --- | --- | --- | --- | --- | --- |
| Sleep Duration | 0.10 | 0.10 | 180.89 | 1.02 | .31 | — |

*Note*. ***p* < .01

**Table 6**

*Between-Person Effects of Subjective Sleep Quality and Duration on Support Mediated by Negative Affect and Perspective-Taking (Study 1)*

| Predictors | | Mediators | *a* | *b* | *ab* | *c* | *c’* | 95% CI  Lower Upper | | |
| --- | --- | --- | --- | --- | --- | --- | --- | --- | --- | --- |
| **Provided Support (Self-Reported)** | | | | | | | | | |  |
| Sleep Quality | NA (and Self-Reported PT) | | -0.58*** | -0.09 | 0.05 | 0.50** | 0.26* | -0.05 | 0.17 | |
| Sleep Duration | NA (and Self-Reported PT) | | -0.17* | -0.28* | 0.05 | -0.03 | 0.01 | 0.001 | 0.08 | |
| Sleep Quality | NA (and Own Perceived PT) | | -0.56*** | -0.05 | 0.03 | 0.50** | 0.16 | -0.07 | 0.13 | |
| Sleep Duration | NA (and Own Perceived PT) | | -0.16* | -0.12 | 0.02 | -0.03 | -0.04 | -0.01 | 0.06 | |
| Sleep Quality | NA (and Partner-Perceived PT) | | -0.62*** | -0.20^†^ | 0.12 | 0.50** | 0.35* | -0.02 | 0.28 | |
| Sleep Duration | NA (and Partner-Perceived PT | | -0.15* | -0.31** | 0.05 | -0.03 | -0.02 | 0.001 | 0.11 | |
| Sleep Quality | Self-Reported PT (and NA) | | 0.11 | 0.65*** | 0.07 | 0.50** | 0.26* | -0.07 | 0.21 | |
| Sleep Duration | Self-Reported PT (and NA) | | -0.11 | 0.65*** | -0.07 | -0.03 | 0.01 | -0.19 | 0.43 | |
| Sleep Quality | Own Perceived PT (and NA) | | 0.27^†^ | 0.61*** | 0.16 | 0.50** | 0.16 | -0.02 | 0.36 | |
| Sleep Duration | Own Perceived PT (and NA) | | -0.04 | 0.62*** | -0.02 | -0.03 | -0.04 | -0.15 | 0.09 | |
| Sleep Quality | Partner-Perceived PT (and NA) | | 0.07 | 0.26*** | 0.02 | 0.50** | 0.35* | -0.06 | 0.11 | |
|  |  | |  |  |  |  |  |  |  | |
| Sleep Duration | Partner-Perceived PT (and NA) | | 0.13 | 0.28** | 0.04 | -0.03 | -0.02 | -0.02 | 0.10 | |
| **Received Support (Partner-Reported)** | | | | | | | | | |  |
| Sleep Quality | | NA (and Self-Reported PT) | -0.58*** | -0.17 | 0.10 | 0.08 | -0.16 | -0.02 | 0.24 | |
| Sleep Duration | | NA (and Self-Reported PT) | -0.17* | -0.22* | 0.04 | 0.10 | 0.11 | 0.10 | 0.01 | |
| Sleep Quality | | NA (and Own Perceived PT) | -0.56*** | -0.13 | 0.07 | 0.08 | -0.24 | -0.08 | 0.12 | |
| Sleep Duration | | NA (and Own Perceived PT) | -0.16* | -0.16 | 0.03 | 0.10 | 0.07 | -0.005 | 0.07 | |
| Sleep Quality | | NA (and Partner-Perceived PT) | -0.62*** | -0.11 | 0.07 | 0.08 | -0.07 | -0.003 | 0.18 | |
| Sleep Duration | | NA (and Partner-Perceived PT) | -0.15* | -0.11 | 0.02 | 0.10 | -0.04 | -0.01 | 0.05 | |
| Sleep Quality | | Self-Reported PT (and NA) | 0.11 | 0.42*** | 0.05 | 0.08 | -0.16 | -0.05 | 0.15 | |
| Sleep Duration | | Self-Reported PT (and NA) | -0.11 | 0.46*** | -0.05 | 0.10 | 0.11 | -0.14 | 0.03 | |
| Sleep Quality | | Own Perceived PT (and NA) | 0.27^†^ | 0.46*** | 0.12 | 0.08 | -0.24 | -0.04 | 0.06 | |
| Sleep Duration | | Own Perceived PT (and NA) | -0.04 | 0.50*** | -0.02 | 0.10 | 0.07 | -0.12 | 0.07 | |
| Sleep Quality | | Partner-Perceived PT (and NA) | 0.07 | 0.73*** | 0.05 | 0.08 | -0.07 | -0.17 | 0.28 | |
| Sleep Duration | | Partner-Perceived PT (and NA) | 0.13 | 0.74*** | 0.10 | 0.10 | -0.04 | -0.05 | 0.24 | |

*Note*. NA = Negative Affect. PT = Perspective-Taking. ^†^*p* < .10, **p* < .05, ***p* < .01, ****p* < .001

**p* < 0.05

**Table 7**

*Between-Person Effects of Subjective Sleep Quality and Sleep Duration on Support Provision (Study 2)*

| Predictors | *b* | *SE* | *df* | *t* | *p* | R^2^ |
| --- | --- | --- | --- | --- | --- | --- |

**Provided Support (Self-Reported)**

**————————————————————————————————————————————————**

| Sleep Quality | 0.14 | 0.11 | 143.09 | 1.26 | .21 | — |
| --- | --- | --- | --- | --- | --- | --- |
| Sleep Duration | 0.01 | 0.06 | 160.82 | 0.25 | .80 | — |

**Provided Support (Partner-Reported)**

**————————————————————————————————————————————————**

| Sleep Quality | 0.17 | 0.11 | 143.10 | 1.52 | .13 | — | |  |
| --- | --- | --- | --- | --- | --- | --- | --- | --- |
| Sleep Duration | 0.07 | 0.06 | 160.75 | 1.26 | .21 | | — | |

**Received Support (Self-Reported)**

**————————————————————————————————————————————————**

| Sleep Quality  Sleep Duration | 0.23^†^  0.03 | 0.12  0.06 | 148.89  169.19 | 1.90  0.45 | .06  .66 | 0.01  — |
| --- | --- | --- | --- | --- | --- | --- |

**Received Support (Partner-Reported)**

**————————————————————————————————————————————————**

| Sleep Quality | 0.06 | 0.12 | 148.91 | 0.51 | .61 | — |
| --- | --- | --- | --- | --- | --- | --- |
| Sleep Duration | 0.05 | 0.06 | 169.11 | 0.78 | .44 | — |

*Note*. ^†^*p* < .10

**Table 8**

*Between-Person Effects of Subjective Sleep Quality and Duration on Support Mediated by Negative Affect and Perspective-Taking (Study 2)*

| Predictors | | Mediators | *a* | *b* | *ab* | *c* | *c’* | 95% CI  Lower Upper | | |
| --- | --- | --- | --- | --- | --- | --- | --- | --- | --- | --- |
| **Provided Support (Self-Reported)** | | | | | | | | | |  |
| Sleep Quality | NA (and Self-Reported PT) | | -0.22** | -0.08 | 0.02 | 0.14 | -0.06 | -0.004 | 0.05 | |
| Sleep Duration | NA (and Self-Reported PT) | | -0.02 | -0.08 | 0.002 | 0.01 | -0.04 | -0.01 | 0.01 | |
| Sleep Quality | NA (and Own Perceived PT) | | -0.23** | -0.19** | 0.04 | 0.14 | -0.11 | 0.01 | 0.09 | |
| Sleep Duration | NA (and Own Perceived PT) | | -0.02 | -0.18** | 0.004 | 0.01 | -0.04 | -0.01 | 0.02 | |
| Sleep Quality | NA (and Partner-Perceived PT) | | -0.23*** | -0.15^†^ | 0.04 | 0.14 | 0.11 | -0.002 | 0.08 | |
| Sleep Duration | NA (and Partner-Perceived PT) | | -0.02 | -0.16* | 0.003 | 0.01 | -0.01 | -0.01 | 0.02 | |
| Sleep Quality | Self-Reported PT (and NA) | | 0.25^†^ | 0.70*** | 0.18 | 0.14 | -0.06 | -0.001 | 0.37 | |
| Sleep Duration | Self-Reported PT (and NA) | | 0.08 | 0.70*** | 0.06 | 0.01 | -0.04 | -0.04 | 0.14 | |
| Sleep Quality | Own Perceived PT (and NA) | | 0.35* | 0.64*** | 0.22 | 0.14 | -0.11 | 0.05 | 0.41 | |
| Sleep Duration | Own Perceived PT (and NA) | | 0.08 | 0.63*** | 0.05 | 0.01 | -0.04 | -0.04 | 0.14 | |
| Sleep Quality | Partner-Perceived PT (and NA) | | -0.04 | 0.57*** | 0.02 | 0.14 | 0.11 | -0.19 | 0.14 | |
|  |  | |  |  |  |  |  |  |  | |
| Sleep Duration | Partner-Perceived PT (and NA) | | 0.02 | 0.57*** | 0.01 | 0.01 | -0.01 | -0.07 | 0.09 | |
| **Received Support (Partner-Reported)** | | | | | | | | | |  |
| Sleep Quality | | NA (and Self-Reported PT) | -0.22** | -0.12 | 0.03 | 0.06 | -0.14 | -0.01 | 0.07 | |
| Sleep Duration | | NA (and Self-Reported PT) | -0.02 | -0.13 | 0.003 | 0.05 | -0.003 | -0.01 | 0.02 | |
| Sleep Quality | | NA (and Own Perceived PT) | -0.23** | -0.14* | 0.03 | 0.06 | -0.20 | 0.002 | 0.08 | |
| Sleep Duration | | NA (and Own Perceived PT) | -0.02 | -0.14* | 0.003 | 0.05 | -0.01 | -0.01 | 0.01 | |
| Sleep Quality | | NA (and Partner-Perceived PT) | -0.23*** | -0.09^†^ | 0.02 | 0.06 | 0.05 | -0.002 | 0.05 | |
| Sleep Duration | | NA (and Partner-Perceived PT) | -0.02 | -0.09^†^ | 0.002 | 0.05 | 0.02 | -0.04 | 0.14 | |
| Sleep Quality | | Self-Reported PT (and NA) | 0.25^†^ | 0.64*** | 0.16 | 0.06 | -0.14 | -0.01 | 0.33 | |
| Sleep Duration | | Self-Reported PT (and NA) | 0.08 | 0.65*** | 0.05 | 0.05 | -0.003 | -0.04 | 0.14 | |
| Sleep Quality | | Own Perceived PT (and NA) | 0.35* | 0.70*** | 0.25 | 0.06 | -0.20 | 0.05 | 0.44 | |
| Sleep Duration | | Own Perceived PT (and NA) | 0.08 | 0.70*** | 0.06 | 0.05 | -0.01 | -0.04 | 0.15 | |
| Sleep Quality | | Partner-Perceived PT (and NA) | -0.04 | 0.75** | -0.03 | 0.06 | 0.05 | -0.25 | 0.18 | |
| Sleep Duration | | Partner-Perceived PT (and NA) | 0.02 | 0.75*** | 0.02 | 0.05 | 0.02 | -0.09 | 0.12 | |

*Note*. NA = Negative Affect. PT = Perspective-Taking. ^†^*p* < .10, **p* < .05, ***p* < .01, ****p* < .001

**p* < 0.05

**Table 9**

*Effects of Sleep Quality (Composite) on Support Provision (Study 1)*

| Predictors | *b* | *SE* | *df* | *t* | *p* | R^2^ |
| --- | --- | --- | --- | --- | --- | --- |

**Provided Support (Self-Reported)**

**————————————————————————————————————————————————**

| WP Sleep Quality | 0.16* | 0.08 | 1065.60 | 2.01 | .045 | 0.002 |
| --- | --- | --- | --- | --- | --- | --- |
| BP Sleep Quality | 0.32 | 0.21 | 193.11 | 1.50 | .13 | — |

**Provided Support (Partner-Reported)**

**————————————————————————————————————————————————**

| WP Sleep Quality | 0.10 | 0.08 | 1063.98 | 1.28 | .20 | — | |  |
| --- | --- | --- | --- | --- | --- | --- | --- | --- |
| BP Sleep Quality | -0.06 | 0.21 | 192.28 | -0.27 | .79 | | — | |

**Received Support (Self-Reported)**

**————————————————————————————————————————————————**

| WP Sleep Quality  BP Sleep Quality | 0.15^†^  0.23 | 0.08  0.21 | 1062.28  189.73 | 1.96  1.08 | .05  .28 | 0.002  — |
| --- | --- | --- | --- | --- | --- | --- |

**Received Support (Partner-Reported)**

**————————————————————————————————————————————————**

| WP Sleep Quality | 0.04 | 0.08 | 1061.72 | 0.47 | .64 | — |
| --- | --- | --- | --- | --- | --- | --- |
| BP Sleep Quality | 0.06 | 0.21 | 188.70 | 0.29 | .77 | — |

WP = Within-Person, BP = Between-Person. *Note*. ^†^*p* < 0.1 **p* < .05

**Table 10**

*Effects of Sleep Quality (Composite) on Support Mediated by Negative Affect and Perspective-Taking (Study 1)*

*Note*. WP = Within-Person, BP = Between-Person, PT = Perspective-Taking. ^†^*p* < .10, **p* < .05, ***p* < .01, ****p* < .001

**p* < 0.05

| Predictors | Mediators | *a* | *b* | *ab* | *c* | *c’* | 95% CI  Lower Upper | | |
| --- | --- | --- | --- | --- | --- | --- | --- | --- | --- |
| **Provided Support (Self-Reported)** | | | | | | | | |  |
| WP Sleep Quality | Negative Affect | -0.43*** | -0.23*** | 0.21 | 0.16* | 0.07 | 0.06 | 0.14 | |
| BP Sleep Quality | Negative Affect | -0.93*** | -0.31** | 0.13 | 0.32 | 0.05 | 0.08 | 0.52 | |
| WP Sleep Quality | Self-Reported PT | 0.11 | 0.40*** | 0.04 | 0.16* | 0.14^†^ | -0.01 | 0.10 | |
| BP Sleep Quality | Self-Reported PT | -0.09 | 0.68*** | -0.06 | 0.32 | 0.31^†^ | -0.32 | 0.21 | |
| WP Sleep Quality | Partner-Perceived PT | 0.05 | 0.08* | 0.004 | 0.16* | 0.15^†^ | -0.01 | 0.02 | |
| BP Sleep Quality | Partner-Perceived PT | 0.25 | 0.31*** | 0.08 | 0.32 | 0.26 | -0.05 | 0.22 | |
| WP Sleep Quality | Own Perceived PT | 0.15^†^ | 0.39*** | 0.06 | 0.16* | 0.11 | 0.0003 | 0.11 | |
|  |  |  |  |  |  |  |  |  | |
| BP Sleep Quality | Own Perceived PT | 0.33 | 0.64*** | 0.21 | 0.32 | 0.05 | -0.06 | 0.49 | |
| **Received Support (Partner-Reported)** | | | | | | | | |  |
| WP Sleep Quality | Negative Affect | -0.43*** | -0.08* | 0.03 | 0.04 | 0.001 | 0.001 | 0.07 | |
| BP Sleep Quality | Negative Affect | -0.93*** | -0.22^†^ | 0.20 | 0.06 | -0.13 | 0.001 | 0.43 | |
| WP Sleep Quality | Self-Reported PT | 0.11 | 0.07* | 0.01 | 0.04 | 0.02 | -0.002 | 0.02 | |
| BP Sleep Quality | Self-Reported PT | -0.09 | 0.45*** | -0.01 | 0.06 | 0.03 | -0.23 | 0.14 | |
| WP Sleep Quality | Partner-Perceived PT | 0.05 | 0.49*** | 0.02 | 0.04 | 0.01 | -0.05 | 0.10 | |
| BP Sleep Quality | Partner-Perceived PT | 0.25 | 0.76*** | 0.19 | 0.06 | -0.12 | -0.14 | 0.51 | |
| WP Sleep Quality | Own Perceived PT | 0.15^†^ | 0.13*** | 0.02 | 0.04 | 0.01 | -0.0001 | 0.04 | |
| BP Sleep Quality | Own Perceived PT | 0.33 | 0.53*** | 0.17 | 0.06 | -0.19 | -0.05 | 0.42 | |

**Table 11**

*Within-Person Effects of Subjective Sleep Quality and Duration on Support Mediated by Negative Affect and Perspective-Taking (Study 1)*

| Predictors | Mediators | *a* | *b* | *ab* | *c* | *c’* | 95% CI  Lower Upper | | |
| --- | --- | --- | --- | --- | --- | --- | --- | --- | --- |
| **Provided Support (Self-Reported)** | | | | | | | | |  |
| Sleep Quality | Negative Affect | -0.22*** | -0.23*** | 0.05 | 0.11* | 0.07 | 0.03 | 0.08 | |
| Sleep Duration | Negative Affect | -0.07*** | -0.24*** | 0.02 | -0.01 | -0.02 | 0.01 | 0.03 | |
| Sleep Quality | Self-Reported PT | 0.10* | 0.40*** | 0.04 | 0.11* | 0.08^†^ | 0.01 | 0.08 | |
| Sleep Duration | Self-Reported PT | 0.01 | 0.07* | 0.001 | -0.01 | -0.03 | -0.003 | 0.01 | |
| Sleep Quality | Partner-Perceived PT | 0.04 | 0.09* | 0.004 | 0.11* | 0.11* | -0.004 | 0.01 | |
| Sleep Duration | Partner-Perceived PT | -0.005 | 0.07* | -0.0004 | -0.01 | -0.01 | -0.004 | 0.004 | |
| Sleep Quality | Own Perceived PT | 0.12** | 0.39*** | 0.05 | 0.11* | 0.07 | 0.01 | 0.08 | |
|  |  |  |  |  |  |  |  |  | |
| Sleep Duration | Own Perceived PT | 0.03 | 0.39*** | 0.01 | -0.01 | -0.02 | -0.01 | 0.03 | |
| **Received Support (Partner-Reported)** | | | | | | | | |  |
| Sleep Quality | Negative Affect | -0.22*** | -0.07^†^ | 0.02 | 0.07 | 0.06 | -0.001 | 0.03 | |
| Sleep Duration | Negative Affect | -0.07*** | -0.04* | 0.003 | -0.02 | -0.03 | 0.0004 | 0.01 | |
| Sleep Quality | Self-Reported PT | 0.10* | 0.08* | 0.01 | 0.07 | 0.06 | 0.0002 | 0.02 | |
| Sleep Duration | Self-Reported PT | 0.01 | 0.07* | 0.001 | -0.02 | -0.03 | -0.003 | 0.01 | |
| Sleep Quality | Partner-Perceived PT | 0.04 | 0.49*** | 0.02 | 0.07 | 0.05 | -0.02 | 0.06 | |
| Sleep Duration | Partner-Perceived PT | -0.005 | 0.49*** | -0.002 | -0.02 | -0.03 | -0.03 | 0.02 | |
| Sleep Quality | Own Perceived PT | 0.12** | 0.13*** | 0.02 | 0.07 | 0.05 | 0.003 | 0.03 | |
| Sleep Duration | Own Perceived PT | 0.03 | 0.13*** | 0.004 | -0.02 | -0.03 | -0.003 | 0.01 | |

*Note*. PT = Perspective-Taking. ^†^*p* < .10, **p* < .05, ***p* < .01, ****p* < .001

**p* < 0.05

**Table 12**

*Between-Person Effects of Subjective Sleep Quality and Duration on Support Mediated by Negative Affect and Perspective-Taking (Study 1)*

| Predictors | Mediators | *a* | *b* | *ab* | *c* | *c’* | 95% CI  Lower Upper | | |
| --- | --- | --- | --- | --- | --- | --- | --- | --- | --- |
| **Provided Support (Self-Reported)** | | | | | | | | |  |
| Sleep Quality | Negative Affect | -0.62*** | -0.19^†^ | 0.12 | 0.50** | 0.38* | -0.02 | 0.27 | |
| Sleep Duration | Negative Affect | -0.16* | -0.33** | 0.05 | -0.03 | -0.08 | 0.004 | 0.12 | |
| Sleep Quality | Self-Reported PT | 0.19 | 0.66*** | 0.13 | 0.50** | 0.32** | -0.04 | 0.30 | |
| Sleep Duration | Self-Reported PT | -0.08 | 0.68*** | -0.05 | -0.03 | 0.04 | -0.17 | 0.07 | |
| Sleep Quality | Partner-Perceived PT | 0.13 | 0.29*** | 0.04 | 0.50** | 0.47*** | -0.04 | 0.12 | |
| Sleep Duration | Partner-Perceived PT | 0.15 | 0.33*** | 0.04 | -0.03 | -0.07 | -0.01 | 0.12 | |
| Sleep Quality | Own Perceived PT | 0.42** | 0.62*** | 0.26 | 0.50** | 0.19^†^ | 0.08 | 0.44 | |
|  |  |  |  |  |  |  |  |  | |
| Sleep Duration | Own Perceived PT | 0.01 | 0.64*** | 0.01 | -0.03 | -0.02 | -0.12 | 0.12 | |
| **Received Support (Partner-Reported)** | | | | | | | | |  |
| Sleep Quality | Negative Affect | -0.62*** | -0.15 | 0.09 | 0.08 | -0.01 | -0.04 | 0.24 | |
| Sleep Duration | Negative Affect | -0.16* | -0.19^†^ | 0.03 | 0.10 | 0.07 | -0.004 | 0.08 | |
| Sleep Quality | Self-Reported PT | 0.19 | 0.42*** | 0.08 | 0.08 | -0.05 | -0.03 | 0.20 | |
| Sleep Duration | Self-Reported PT | -0.08 | 0.46*** | -0.04 | 0.10 | 0.13 | -0.12 | 0.05 | |
| Sleep Quality | Partner-Perceived PT | 0.13 | 0.73*** | 0.09 | 0.08 | -0.01 | -0.11 | 0.29 | |
| Sleep Duration | Partner-Perceived PT | 0.15 | 0.75*** | 0.11 | 0.10 | -0.02 | -0.03 | 0.26 | |
| Sleep Quality | Own Perceived PT | 0.42** | 0.47*** | 0.20 | 0.08 | -0.17 | 0.06 | 0.35 | |
| Sleep Duration | Own Perceived PT | 0.01 | 0.53*** | 0.01 | 0.10 | 0.10 | -0.10 | 0.10 | |

*Note*. PT = Perspective-Taking. ^†^*p* < .10, **p* < .05, ***p* < .01, ****p* < .001

**p* < 0.05

**Table 13**

*Effects of Sleep Quality (Composite) on Support Provision (Study 2)*

| Predictors | *b* | *SE* | *df* | *t* | *p* | R^2^ |
| --- | --- | --- | --- | --- | --- | --- |

**Provided Support (Self-Reported)**

**————————————————————————————————————————————————**

| WP Sleep Quality | 0.03 | 0.02 | 2170.04 | 1.60 | .11 | — |
| --- | --- | --- | --- | --- | --- | --- |
| BP Sleep Quality | 0.11 | 0.12 | 148.37 | 0.97 | .33 | — |

**Provided Support (Partner-Reported)**

**————————————————————————————————————————————————**

| WP Sleep Quality | 0.002 | 0.02 | 2170.01 | 0.13 | .90 | — | |  |
| --- | --- | --- | --- | --- | --- | --- | --- | --- |
| BP Sleep Quality | 0.18 | 0.12 | 148.37 | 1.58 | .12 | | — | |

**Received Support (Self-Reported)**

**————————————————————————————————————————————————**

| WP Sleep Quality  BP Sleep Quality | 0.01  0.18 | 0.02  0.12 | 2134.82  155.45 | 0.51  1.49 | .61  .14 | —  — |
| --- | --- | --- | --- | --- | --- | --- |

**Received Support (Partner-Reported)**

**————————————————————————————————————————————————**

| WP Sleep Quality | 0.06** | 0.02 | 2134.82 | 2.81 | .005 | 0.004 |
| --- | --- | --- | --- | --- | --- | --- |
| BP Sleep Quality | 0.08 | 0.12 | 155.44 | 0.69 | .49 | — |

WP = Within-Person, BP = Between-Person. *Note*. ***p* < 0.01

**Table 14**

*Effects of Sleep Quality (Composite) on Support Mediated by Negative Affect and Perspective-Taking (Study 2)*

*Note*. WP = Within-Person, BP = Between-Person, PT = Perspective-Taking. ^†^*p* < .10, **p* < .05, ***p* < .01, ****p* < .001

**p* < 0.05

| Predictors | Mediators | *a* | *b* | *ab* | *c* | *c’* | 95% CI  Lower Upper | | |
| --- | --- | --- | --- | --- | --- | --- | --- | --- | --- |
| **Provided Support (Self-Reported)** | | | | | | | | |  |
| WP Sleep Quality | Negative Affect | -0.08*** | -0.19*** | 0.02 | 0.03 | 0.01 | 0.01 | 0.02 | |
| BP Sleep Quality | Negative Affect | -0.16* | -0.15 | 0.02 | 0.11 | 0.09 | -0.01 | 0.08 | |
| WP Sleep Quality | Self-Reported PT | 0.02 | 0.40*** | 0.01 | 0.03 | 0.02 | -0.01 | 0.03 | |
| BP Sleep Quality | Self-Reported PT | 0.27* | 0.70*** | 0.19 | 0.11 | -0.07 | 0.002 | 0.37 | |
| WP Sleep Quality | Partner-Perceived PT | 0.08** | 0.16*** | 0.01 | 0.03 | 0.01 | 0.004 | 0.02 | |
| BP Sleep Quality | Partner-Perceived PT | 0.01 | 0.56*** | 0.01 | 0.11 | 0.09 | -0.15 | 0.16 | |
| WP Sleep Quality | Own Perceived PT | 0.03 | 0.29*** | 0.01 | 0.03 | 0.02 | -0.01 | 0.02 | |
|  |  |  |  |  |  |  |  |  | |
| BP Sleep Quality | Own Perceived PT | 0.30 | 0.63*** | 0.19 | 0.11 | -0.08 | 0.02 | 0.37 | |
| **Received Support (Partner-Reported)** | | | | | | | | |  |
| WP Sleep Quality | Negative Affect | -0.08*** | -0.12*** | 0.01 | 0.06** | 0.05* | 0.004 | 0.02 | |
| BP Sleep Quality | Negative Affect | -0.16* | -0.16 | 0.03 | 0.08 | 0.06 | -0.01 | 0.08 | |
| WP Sleep Quality | Self-Reported PT | 0.02 | 0.10*** | 0.002 | 0.06** | 0.06** | -0.003 | 0.01 | |
| BP Sleep Quality | Self-Reported PT | 0.27* | 0.65*** | 0.18 | 0.08 | -0.08 | 0.004 | 0.35 | |
| WP Sleep Quality | Partner-Perceived PT | 0.08** | 0.50*** | 0.04 | 0.06** | 0.02 | 0.01 | 0.07 | |
| BP Sleep Quality | Partner-Perceived PT | 0.01 | 0.76*** | 0.01 | 0.08 | 0.07 | -0.20 | 0.23 | |
| WP Sleep Quality | Own Perceived PT | 0.03 | 0.20*** | 0.01 | 0.06** | 0.05 | -0.004 | 0.02 | |
| BP Sleep Quality | Own Perceived PT | 0.30 | 0.70*** | 0.21 | 0.08 | -0.13 | 0.02 | 0.41 | |

**Table 15**

*Within-Person Effects of Subjective Sleep Quality and Duration on Support Mediated by Negative Affect and Perspective-Taking (Study 2)*

*Note*. PT = Perspective-Taking. ^†^*p* < .10, **p* < .05, ***p* < .01, ****p* < .001

**p* < 0.05

| Predictors | Mediators | *a* | *b* | *ab* | *c* | *c’* | 95% CI  Lower Upper | | |
| --- | --- | --- | --- | --- | --- | --- | --- | --- | --- |
| **Provided Support (Self-Reported)** | | | | | | | | |  |
| Sleep Quality | Negative Affect | -0.09*** | -0.19*** | 0.02 | 0.04* | 0.02 | 0.01 | 0.03 | |
| Sleep Duration | Negative Affect | -0.01 | -0.20*** | 0.002 | 0.001 | -0.002 | -0.001 | 0.01 | |
| Sleep Quality | Self-Reported PT | 0.03 | 0.40*** | 0.01 | 0.04* | 0.03* | -0.01 | 0.03 | |
| Sleep Duration | Self-Reported PT | 0.002 | 0.40*** | 0.001 | 0.001 | 0.001 | -0.01 | 0.01 | |
| Sleep Quality | Partner-Perceived PT | 0.06* | 0.16*** | 0.01 | 0.04* | 0.03^†^ | 0.001 | 0.02 | |
| Sleep Duration | Partner-Perceived PT | 0.04** | 0.16*** | 0.01 | 0.001 | -0.005 | 0.002 | 0.01 | |
| Sleep Quality | Own Perceived PT | 0.03 | 0.29*** | 0.01 | 0.04* | 0.03* | -0.01 | 0.02 | |
|  |  |  |  |  |  |  |  |  | |
| Sleep Duration | Own Perceived PT | 0.04 | 0.29*** | 0.01 | 0.001 | -0.002 | -0.005 | 0.01 | |
| **Received Support (Partner-Reported)** | | | | | | | | |  |
| Sleep Quality | Negative Affect | -0.09*** | -0.12*** | 0.01 | 0.06** | 0.04* | 0.01 | 0.02 | |
| Sleep Duration | Negative Affect | -0.01 | -0.12*** | 0.001 | 0.02^†^ | 0.02^†^ | -0.001 | 0.004 | |
| Sleep Quality | Self-Reported PT | 0.03 | 0.10*** | 0.003 | 0.06** | 0.05** | -0.002 | 0.01 | |
| Sleep Duration | Self-Reported PT | 0.002 | 0.10*** | 0.0002 | 0.02^†^ | 0.02^†^ | -0.002 | 0.003 | |
| Sleep Quality | Partner-Perceived PT | 0.06* | 0.50*** | 0.03 | 0.06** | 0.03^†^ | 0.004 | 0.05 | |
| Sleep Duration | Partner-Perceived PT | 0.04** | 0.50*** | 0.02 | 0.02^†^ | 0.001 | 0.01 | 0.03 | |
| Sleep Quality | Own Perceived PT | 0.03 | 0.19*** | 0.01 | 0.06** | 0.05* | -0.004 | 0.02 | |
| Sleep Duration | Own Perceived PT | 0.04 | 0.20*** | 0.01 | 0.02^†^ | 0.02^†^ | -0.003 | 0.01 | |

**Table 16**

*Between-Person Effects of Subjective Sleep Quality and Duration on Support Mediated by Negative Affect and Perspective-Taking (Study 2)*

*Note*. PT = Perspective-Taking. ^†^*p* < .10, **p* < .05, ***p* < .01, ****p* < .001

**p* < 0.05

| Predictors | Mediators | *a* | *b* | *ab* | *c* | *c’* | 95% CI  Lower Upper | | |
| --- | --- | --- | --- | --- | --- | --- | --- | --- | --- |
| **Provided Support (Self-Reported)** | | | | | | | | |  |
| Sleep Quality | Negative Affect | -0.23** | -0.14 | 0.03 | 0.14 | 0.11 | -0.01 | 0.09 | |
| Sleep Duration | Negative Affect | -0.02 | -0.16 | 0.003 | 0.01 | 0.01 | -0.01 | 0.02 | |
| Sleep Quality | Self-Reported PT | 0.27* | 0.70*** | 0.19 | 0.14 | -0.05 | 0.01 | 0.37 | |
| Sleep Duration | Self-Reported PT | 0.08 | 0.70*** | 0.06 | 0.01 | -0.04 | -0.04 | 0.15 | |
| Sleep Quality | Partner-Perceived PT | -0.02 | 0.56*** | -0.01 | 0.14 | 0.15 | -0.16 | 0.14 | |
| Sleep Duration | Partner-Perceived PT | 0.02 | 0.56*** | 0.01 | 0.01 | -0.01 | -0.09 | 0.12 | |
| Sleep Quality | Own Perceived PT | 0.33* | 0.64*** | 0.21 | 0.14 | -0.07 | 0.03 | 0.39 | |
|  |  |  |  |  |  |  |  |  | |
| Sleep Duration | Own Perceived PT | 0.08 | 0.63*** | 0.05 | 0.01 | -0.04 | -0.04 | 0.14 | |
| **Received Support (Partner-Reported)** | | | | | | | | |  |
| Sleep Quality | Negative Affect | -0.23** | -0.16 | 0.04 | 0.06 | 0.02 | -0.01 | 0.10 | |
| Sleep Duration | Negative Affect | -0.02 | -0.16 | 0.003 | 0.05 | 0.04 | -0.01 | 0.02 | |
| Sleep Quality | Self-Reported PT | 0.27* | 0.64*** | 0.17 | 0.06 | -0.11 | 0.01 | 0.35 | |
| Sleep Duration | Self-Reported PT | 0.08 | 0.65*** | 0.05 | 0.05 | -0.0001 | -0.03 | 0.14 | |
| Sleep Quality | Partner-Perceived PT | -0.02 | 0.76*** | -0.02 | 0.06 | 0.07 | -0.23 | 0.20 | |
| Sleep Duration | Partner-Perceived PT | 0.02 | 0.75*** | 0.02 | 0.05 | 0.03 | -0.09 | 0.11 | |
| Sleep Quality | Own Perceived PT | 0.33* | 0.70*** | 0.23 | 0.06 | -0.16 | 0.04 | 0.43 | |
| Sleep Duration | Own Perceived PT | 0.08 | 0.70*** | 0.06 | 0.05 | -0.01 | -0.04 | 0.15 | |

**Table 17**

*Within-Person Effects of Subjective Sleep Quality and Duration on Support Mediated by Perspective-Taking Item Two (Study 2)*

| Predictors | Mediators | *a* | *b* | *ab* | *c* | *c’* | 95% CI  Lower Upper | | |
| --- | --- | --- | --- | --- | --- | --- | --- | --- | --- |
| **Provided Support (Self-Reported)** | | | | | | | | |  |
| Sleep Quality | Self-Reported PT | -0.01 | 0.17*** | -0.002 | 0.04* | 0.04* | -0.003 | 0.0001 | |
| Sleep Duration | Self-Reported PT | -0.001 | 0.17*** | -0.0002 | 0.001 | 0.001 | -0.005 | 0.004 | |
| Sleep Quality | Partner-Perceived PT | -0.01 | 0.09*** | -0.001 | 0.04* | 0.04* | -0.01 | 0.004 | |
| Sleep Duration | Partner-Perceived PT | -0.001 | 0.09*** | -0.0001 | 0.001 | 0.001 | -0.003 | 0.003 | |
| Sleep Quality | Own Perceived PT | -0.004 | 0.14*** | -0.001 | 0.04* | 0.04* | -0.01 | 0.01 | |
|  |  |  |  |  |  |  |  |  | |
| Sleep Duration | Own Perceived PT | -0.02 | 0.14*** | -0.003 | 0.001 | 0.004 | -0.01 | 0.002 | |
| **Received Support (Partner-Reported)** | | | | | | | | |  |
| Sleep Quality | Self-Reported PT | -0.01 | 0.04** | -0.0004 | 0.06** | 0.06** | -0.001 | 0.0001 | |
| Sleep Duration | Self-Reported PT | -0.001 | 0.04* | -0.00004 | 0.02^†^ | 0.02^†^ | -0.001 | 0.001 | |
| Sleep Quality | Partner-Perceived PT | -0.01 | 0.24*** | -0.002 | 0.06** | 0.06** | -0.01 | 0.01 | |
| Sleep Duration | Partner-Perceived PT | -0.001 | 0.24*** | -0.0002 | 0.02^†^ | 0.02* | -0.01 | 0.01 | |
| Sleep Quality | Own Perceived PT | -0.004 | 0.03^†^ | -0.0001 | 0.06** | 0.06** | -0.002 | 0.001 | |
| Sleep Duration | Own Perceived PT | -0.02 | 0.03^†^ | -0.001 | 0.02^†^ | 0.02^†^ | -0.002 | 0.0004 | |

*Note*. PT = Perspective-Taking. ^†^*p* < .10, **p* < .05, ***p* < .01, ****p* < .001

**p* < 0.05

**Table 18**

*Between-Person Effects of Subjective Sleep Quality and Duration on Support Mediated by Perspective-Taking Item Two (Study 2)*

| Predictors | Mediators | *a* | *b* | *ab* | *c* | *c’* | 95% CI  Lower Upper | | |
| --- | --- | --- | --- | --- | --- | --- | --- | --- | --- |
| **Provided Support (Self-Reported)** | | | | | | | | |  |
| Sleep Quality | Self-Reported PT | 0.26^†^ | 0.50*** | 0.13 | 0.14 | 0.004 | -0.01 | 0.28 | |
| Sleep Duration | Self-Reported PT | 0.08 | 0.50*** | 0.04 | 0.01 | 0.03 | -0.03 | 0.11 | |
| Sleep Quality | Partner-Perceived PT | 0.02 | -0.10^†^ | -0.002 | 0.14 | 0.14 | -0.04 | 0.03 | |
| Sleep Duration | Partner-Perceived PT | 0.05 | -0.09^†^ | -0.005 | 0.01 | 0.02 | -0.02 | 0.01 | |
| Sleep Quality | Own Perceived PT | 0.36* | 0.49*** | 0.18 | 0.14 | -0.04 | 0.03 | 0.32 | |
|  |  |  |  |  |  |  |  |  | |
| Sleep Duration | Own Perceived PT | 0.12 | 0.48*** | 0.06 | 0.01 | -0.04 | -0.01 | 0.13 | |
| **Received Support (Partner-Reported)** | | | | | | | | |  |
| Sleep Quality | Self-Reported PT | 0.26^†^ | 0.13* | 0.04 | 0.06 | 0.02 | -0.004 | 0.09 | |
| Sleep Duration | Self-Reported PT | 0.08 | 0.12* | 0.01 | 0.05 | 0.04 | -0.01 | 0.03 | |
| Sleep Quality | Partner-Perceived PT | 0.02 | 0.59*** | 0.01 | 0.06 | 0.05 | -0.16 | 0.19 | |
| Sleep Duration | Partner-Perceived PT | 0.05 | 0.60*** | 0.03 | 0.05 | 0.02 | -0.06 | 0.12 | |
| Sleep Quality | Own Perceived PT | 0.36* | 0.40*** | 0.14 | 0.06 | -0.09 | 0.02 | 0.27 | |
| Sleep Duration | Own Perceived PT | 0.12 | 0.41*** | 0.05 | 0.05 | -0.001 | -0.01 | 0.11 | |

*Note*. PT = Perspective-Taking. ^†^*p* < .10, **p* < .05, ***p* < .01, ****p* < .001

**p* < 0.05

**Table 19**

*Within-Person Effects of Subjective Sleep Quality and Duration on Support Mediated by Perspective-Taking Reverse-Coded Item (Study 2)*

| Predictors | Mediators | *a* | *b* | *ab* | *c* | *c’* | 95% CI  Lower Upper | | |
| --- | --- | --- | --- | --- | --- | --- | --- | --- | --- |
| **Provided Support (Self-Reported)** | | | | | | | | |  |
| Sleep Quality | Self-Reported PT | 0.02 | 0.12*** | 0.002 | 0.04* | 0.04* | -0.003 | 0.01 | |
| Sleep Duration | Self-Reported PT | -0.02 | 0.12** | -0.002 | 0.001 | 0.003 | -0.01 | 0.001 | |
| Sleep Quality | Partner-Perceived PT | 0.07** | 0.09*** | 0.01 | 0.04* | 0.03* | 0.002 | 0.01 | |
| Sleep Duration | Partner-Perceived PT | 0.01 | 0.09*** | 0.001 | 0.001 | -0.003 | -0.002 | 0.003 | |
| Sleep Quality | Own Perceived PT | 0.08** | 0.10*** | 0.01 | 0.04* | 0.03^†^ | 0.003 | 0.01 | |
|  |  |  |  |  |  |  |  |  | |
| Sleep Duration | Own Perceived PT | 0.02 | 0.10*** | 0.002 | 0.001 | -0.001 | -0.001 | 0.005 | |
| **Received Support (Partner-Reported)** | | | | | | | | |  |
| Sleep Quality | Self-Reported PT | 0.02 | 0.05** | 0.001 | 0.06** | 0.05** | -0.001 | 0.004 | |
| Sleep Duration | Self-Reported PT | -0.02 | 0.05** | -0.001 | 0.02^†^ | 0.02* | -0.002 | 0.0005 | |
| Sleep Quality | Partner-Perceived PT | 0.07** | 0.15*** | 0.01 | 0.06** | 0.05* | 0.003 | 0.02 | |
| Sleep Duration | Partner-Perceived PT | 0.01 | 0.15*** | 0.002 | 0.02^†^ | 0.02^†^ | -0.003 | 0.01 | |
| Sleep Quality | Own Perceived PT | 0.08** | 0.06*** | 0.005 | 0.06** | 0.05* | 0.002 | 0.01 | |
| Sleep Duration | Own Perceived PT | 0.02 | 0.06*** | 0.001 | 0.02^†^ | 0.02^†^ | -0.001 | 0.003 | |

*Note*. PT = Perspective-Taking. ^†^*p* < .10, **p* < .05, ***p* < .01, ****p* < .001

**p* < 0.05

**Table 20**

*Between-Person Effects of Subjective Sleep Quality and Duration on Support Mediated by Perspective-Taking Reverse-Coded Item (Study 2)*

| Predictors | Mediators | *a* | *b* | *ab* | *c* | *c’* | 95% CI  Lower Upper | | |
| --- | --- | --- | --- | --- | --- | --- | --- | --- | --- |
| **Provided Support (Self-Reported)** | | | | | | | | |  |
| Sleep Quality | Self-Reported PT | 0.14 | 0.24** | 0.03 | 0.14 | 0.11 | -0.01 | 0.09 | |
| Sleep Duration | Self-Reported PT | 0.04 | 0.24** | 0.01 | 0.01 | 0.004 | -0.01 | 0.04 | |
| Sleep Quality | Partner-Perceived PT | 0.02 | 0.11 | 0.002 | 0.14 | 0.14 | -0.03 | 0.03 | |
| Sleep Duration | Partner-Perceived PT | -0.01 | 0.12 | 0.001 | 0.01 | 0.02 | -0.02 | 0.01 | |
| Sleep Quality | Own Perceived PT | 0.28** | 0.24** | 0.07 | 0.14 | 0.08 | 0.01 | 0.14 | |
|  |  |  |  |  |  |  |  |  | |
| Sleep Duration | Own Perceived PT | 0.08 | 0.24** | 0.02 | 0.01 | -0.01 | -0.005 | 0.05 | |
| **Received Support (Partner-Reported)** | | | | | | | | |  |
| Sleep Quality | Self-Reported PT | 0.14 | 0.15^†^ | 0.02 | 0.06 | 0.04 | -0.01 | 0.07 | |
| Sleep Duration | Self-Reported PT | 0.04 | 0.15^†^ | 0.01 | 0.05 | 0.04 | -0.01 | 0.03 | |
| Sleep Quality | Partner-Perceived PT | 0.02 | 0.30*** | 0.01 | 0.06 | 0.05 | -0.06 | 0.07 | |
| Sleep Duration | Partner-Perceived PT | -0.01 | 0.33*** | -0.003 | 0.05 | 0.05 | -0.04 | 0.03 | |
| Sleep Quality | Own Perceived PT | 0.28** | 0.18* | 0.05 | 0.06 | 0.01 | 0.01 | 0.09 | |
| Sleep Duration | Own Perceived PT | 0.08 | 0.16* | 0.01 | 0.05 | 0.03 | -0.003 | 0.04 | |

*Note*. PT = Perspective-Taking. ^†^*p* < .10, **p* < .05, ***p* < .01, ****p* < .001

**p* < 0.05

**Table 21**

*Within-Person Effects of Subjective Sleep Quality and Duration on Negative Affect Mediated by Support (Study 1)*

| Predictors | | Mediators | 95% CI  Lower Upper | |
| --- | --- | --- | --- | --- |
| Sleep Quality | Self-Reported Support (and Self-Reported PT) | | -0.02 | 0.01 |
| Sleep Duration | Self-Reported Support (and Self-Reported PT) | | -0.004 | 0.01 |
| Sleep Quality | Self-Reported Support (and Own Perceived PT) | | -0.02 | 0.01 |
| Sleep Duration | Self-Reported Support (and Own Perceived PT) | | -0.005 | 0.01 |
| Sleep Quality | Self-Reported Support (and Partner-Perceived PT) | | -0.02 | 0.01 |
| Sleep Duration | Self-Reported Support (and Partner-Perceived PT) | | -0.004 | 0.01 |
| Sleep Quality | Partner-Perceived Support (and Self-Reported PT) | | -0.01 | 0.002 |
| Sleep Duration | Partner-Perceived Support (and Self-Reported PT) | | -0.001 | 0.01 |
| Sleep Quality | Partner-Perceived Support (and Own Perceived PT) | | -0.01 | 0.004 |
| Sleep Duration | Partner-Perceived Support (and Own Perceived PT) | | -0.003 | 0.01 |
| Sleep Quality | Partner-Perceived Support (and Partner-Perceived PT) | | -0.02 | 0.01 |
|  |  | |  |  |
| Sleep Duration | Partner-Perceived Support (and Partner-Perceived PT) | | -0.001 | 0.01 |

*Note*. PT = Perspective-Taking.

**p* < 0.05

**Table 22**

*Within-Person Effects of Subjective Sleep Quality and Duration on Perspective-Taking Mediated by Support (Study 1)*

| Predictors | | Mediators | 95% CI  Lower Upper | |
| --- | --- | --- | --- | --- |
|  | **Self-Reported Perspective-Taking** | |  |  |
| Sleep Quality | Self-Reported Support (and Negative Affect) | | -0.02 | 0.04 |
| Sleep Duration | Self-Reported Support (and Negative Affect) | | -0.02 | 0.01 |
| Sleep Quality | Partner-Perceived Support (and Negative Affect) | | -0.01 | 0.02 |
| Sleep Duration | Partner-Perceived Support (and Negative Affect) | | -0.01 | 0.002 |
|  | **Own Perceived Perspective-Taking** | |  |  |
| Sleep Quality | Self-Reported Support (and Negative Affect) | | -0.02 | 0.03 |
| Sleep Duration | Self-Reported Support (and Negative Affect) | | -0.02 | 0.01 |
| Sleep Quality | Partner-Perceived Support (and Negative Affect) | | -0.005 | 0.02 |
| Sleep Duration | Partner-Perceived Support (and Negative Affect) | | -0.01 | 0.004 |
|  | **Partner-Perceived Perspective-Taking** | |  |  |
| Sleep Quality | Self-Reported Support (and Negative Affect) | | -0.02 | 0.03 |
| Sleep Duration | Self-Reported Support (and Negative Affect) | | -0.005 | 0.02 |
| Sleep Quality | Partner-Perceived Support (and Negative Affect) | | -0.01 | 0.02 |
|  |  | |  |  |
| Sleep Duration | Partner-Perceived Support (and Negative Affect) | | -0.01 | 0.002 |

*Note*. PT = Perspective-Taking.

**p* < 0.05

**Table 23**

*Within-Person Effects of Subjective Sleep Quality and Duration on Negative Affect Mediated by Support (Study 2)*

| Predictors | | Mediators | 95% CI  Lower Upper | |
| --- | --- | --- | --- | --- |
| Sleep Quality | Self-Reported Support (and Self-Reported PT) | | -0.01 | 0.003 |
| Sleep Duration | Self-Reported Support (and Self-Reported PT) | | -0.003 | 0.004 |
| Sleep Quality | Self-Reported Support (and Own Perceived PT) | | -0.01 | 0.003 |
| Sleep Duration | Self-Reported Support (and Own Perceived PT) | | -0.004 | 0.005 |
| Sleep Quality | Self-Reported Support (and Partner-Perceived PT) | | -0.01 | 0.004 |
| Sleep Duration | Self-Reported Support (and Partner-Perceived PT) | | -0.003 | 0.01 |
| Sleep Quality | Partner-Perceived Support (and Self-Reported PT) | | -0.01 | -0.0004 |
| Sleep Duration | Partner-Perceived Support (and Self-Reported PT) | | -0.004 | 0.0002 |
| Sleep Quality | Partner-Perceived Support (and Own Perceived PT) | | -0.01 | 0.0002 |
| Sleep Duration | Partner-Perceived Support (and Own Perceived PT) | | -0.004 | 0.0003 |
| Sleep Quality | Partner-Perceived Support (and Partner-Perceived PT) | | -0.01 | 0.0004 |
|  |  | |  |  |
| Sleep Duration | Partner-Perceived Support (and Partner-Perceived PT) | | -0.003 | 0.01 |

*Note*. PT = Perspective-Taking.

**p* < 0.05

**Table 24**

*Within-Person Effects of Subjective Sleep Quality and Duration on Perspective-Taking Mediated by Support (Study 2)*

| Predictors | | Mediators | 95% CI  Lower Upper | |
| --- | --- | --- | --- | --- |
|  | **Self-Reported Perspective-Taking** | |  |  |
| Sleep Quality | Self-Reported Support (and Negative Affect) | | -0.01 | 0.03 |
| Sleep Duration | Self-Reported Support (and Negative Affect) | | -0.01 | 0.01 |
| Sleep Quality | Partner-Perceived Support (and Negative Affect) | | 0.001 | 0.02 |
| Sleep Duration | Partner-Perceived Support (and Negative Affect) | | -0.001 | 0.01 |
|  | **Own Perceived Perspective-Taking** | |  |  |
| Sleep Quality | Self-Reported Support (and Negative Affect) | | -0.01 | 0.03 |
| Sleep Duration | Self-Reported Support (and Negative Affect) | | -0.01 | 0.01 |
| Sleep Quality | Partner-Perceived Support (and Negative Affect) | | -0.001 | 0.04 |
| Sleep Duration | Partner-Perceived Support (and Negative Affect) | | -0.001 | 0.01 |
|  | **Partner-Perceived Perspective-Taking** | |  |  |
| Sleep Quality | Self-Reported Support (and Negative Affect) | | -0.01 | 0.04 |
| Sleep Duration | Self-Reported Support (and Negative Affect) | | -0.01 | 0.02 |
| Sleep Quality | Partner-Perceived Support (and Negative Affect) | | -0.01 | 0.04 |
|  |  | |  |  |
| Sleep Duration | Partner-Perceived Support (and Negative Affect) | | -0.004 | 0.01 |

*Note*. PT = Perspective-Taking.

**p* < 0.05

**Figure 1**

*The Effects of Sleep Quality (Composite) on Partner-Reported Support Provision Moderated by Gender (Lab Study—Change Conversation).*

*Note.* Low values represent 1 SD below the mean, high values represent 1 SD above the mean.

**Figure 2**

*The Effects of Sleep Quality (Composite) on Own Self-Reported Support Provision Moderated by Relationship Length (Lab Study—Distress Conversation).*

*Note.* Low values represent 1 SD below the mean, high values represent 1 SD above the mean.

**Figure 3**

*The Effects of Between-Person Subjective Sleep Quality on Provided Support (Self-Reported) Moderated by Gender (Study 1).*

*Note.* Low values represent 1 SD below the mean, high values represent 1 SD above the mean.

**Figure 4**

*The Effects of Between-Person Subjective Sleep Quality on Received Support (Self-Reported) Moderated by Gender (Study 1).*

*Note.* Low values represent 1 SD below the mean, high values represent 1 SD above the mean.

**Figure 5**

*The Effects of Between-Person Sleep Duration on Provided Support (Self-Reported) Moderated by Gender (Study 1).*

*Note.* Low values represent 1 SD below the mean, high values represent 1 SD above the mean.

**Figure 6**

*The Effects of Between-Person Sleep Duration on Received Support (Self-Reported) Moderated by Gender (Study 1).*

*Note.* Low values represent 1 SD below the mean, high values represent 1 SD above the mean.

**Figure 7**

*The Effects of Between-Person Subjective Sleep Quality on Provided Support (Partner-Reported) Moderated by Relationship Length (Study 1).*

*Note.* Low values represent 1 SD below the mean, high values represent 1 SD above the mean.

**Figure 8**

*The Effects of Within-Person Subjective Sleep Quality on Received Support (Self-Reported) Moderated by Relationship Length (Study 1).*

*Note.* Low values represent 1 SD below the mean, high values represent 1 SD above the mean.

**Figure 9**

*The Effects of Within-Person Sleep Duration on Received Support (Partner-Reported) Moderated by Relationship Length (Study 2).*

*Note.* Low values represent 1 SD below the mean, high values represent 1 SD above the mean.

**Figure 10**

*The Effects of Sleep Quality (Composite) on Self-Reported Support Provision Moderated by Communal Strength (Lab Study—Change Conversation).*

*Note.* Low values represent 1 SD below the mean, high values represent 1 SD above the mean.

**Figure 11**

*The Effects of Sleep Quality (Composite) on Partner-Reported Support Provision Moderated by Communal Strength (Lab Study—Change Conversation).*

*Note.* Low values represent 1 SD below the mean, high values represent 1 SD above the mean.

1. Couples engaged in a neutral conversation, two conversations about distress, two conversations about partner change, and two conversations about gratitude. However, only the conversations about distress and change are analyzed in this study. [↑](#footnote-ref-1)
2. Some participants entered a range of hours for our sleep duration measure. In these cases, we averaged across the range and used the obtained value to represent their overall sleep duration. Moreover, some participants entered a single value (e.g., 23 hours) that made it difficult to deduce whether they were referring to daily or weekly sleep duration and, as such, were removed from analyses. [↑](#footnote-ref-2)
3. We only measured perspective-taking for the distress discussion and, as such, did not run any analyses involving perspective-taking for the change conversation. [↑](#footnote-ref-3)
4. We only measured observer-rated support provision for the distress discussion and, as such, did not run any analyses involving observer-rated support provision for the change conversation. [↑](#footnote-ref-4)
5. Results did not largely differ when the individual subjective sleep quality and sleep duration items were used as predictors instead of the sleep quality composite (see Tables 1 and 2). [↑](#footnote-ref-5)
6. Results did not largely differ when the individual subjective sleep quality and sleep duration items were used as predictors instead of the sleep quality composite (see Tables 3 and 4). [↑](#footnote-ref-6)
7. Results did not largely differ when the individual subjective sleep quality and sleep duration items were used as predictors instead of the sleep quality composite (see Tables 3 and 4). [↑](#footnote-ref-7)
